# Interprotomer crosstalk in mosaic viral glycoprotein trimers provides insight into polyvalent immunogen co-assembly

**DOI:** 10.1371/journal.ppat.1013143

**Published:** 2025-09-22

**Authors:** Chengbo Chen, Klaus N. Lovendahl, Julie M. Overbaugh, Kelly K. Lee

**Affiliations:** 1 Department of Medicinal Chemistry, University of Washington, Seattle, Washington, United States of America; 2 Biological Physics Structure and Design Graduate Program, University of Washington, Seattle, Washington, United States of America; 3 Human Biology Division, Fred Hutchinson Cancer Center, Seattle, Washington, United States of America; Tufts University School of Medicine, UNITED STATES OF AMERICA

## Abstract

SARS-CoV-2 variants have demonstrated the ability to evade immune responses, leading to waves of infection throughout the pandemic. In response, bivalent mRNA vaccines, encoding the original Wuhan-Hu-1 and emerging variants, were developed to display both spike antigens. To date, it has not been determined whether co-transfection and co-translation of different SARS-CoV-2 variants results in co-assembly of mosaic heterotrimer antigens and how this may affect trimer stability, dynamics, and antigenicity. Understanding whether such mosaic heterotrimers can form and their implications for antigen structure can provide important information to guide future polyvalent vaccine design where multiple variants of an antigen are co-formulated. To investigate this, we purified mosaic spike assemblies of both genetically close (Omicron BA.2 and XBB) and distant (Omicron BA.2 and Wuhan-Hu-1 G614) strains. We found that the stability and integrity of mosaic spike trimers were maintained without misfolding or aggregation. Glycosylation profiles likewise were preserved relative to the homotrimer counterparts. Hydrogen/deuterium-exchange mass spectrometry and biolayer-interferometry were used to investigate the mosaic spike dynamics and any impact on epitope presentation and receptor binding. The Omicron-XBB heterotrimer, sharing a common fusion subunit sequence, retained protomer-specific dynamics similar to the corresponding homotrimers in antigenically important regions. The Omicron-G614 heterotrimer, co-assembling from protomers of divergent fusion subunit sequences, likewise showed overall similar dynamics and conformations in the receptor-binding subunit compared to the homotrimers. However, the incorporation of the Wuhan-Hu-1 G614 protomer led to a stabilizing effect on the relatively unstable Omicron fusion subunit in the heterotrimer. These findings reveal structural dynamic crosstalk in mosaic trimers, suggesting a potential for enhanced immunogen display and important considerations to be aware of in the use of polyvalent nucleic acid vaccines.

## Author summary

Here we investigated the possibility of immunogen co-assembly from bivalent nucleic acid vaccines with a focus on probing potential impacts on antigen stability and epitope display. We purified mosaic heterotrimers composed of Omicron BA.2 and Wuhan-Hu-1 G614 protomers as well as those composed of Omicron BA.2 and XBB protomers. Both mosaic heterotrimers maintained their structural integrity, morphology, and N-glycosylation profiles. Using hydrogen/deuterium-exchange mass spectrometry, we demonstrated that both types of heterotrimers preserved strain-specific dynamics in critical antigenic regions, comparable to their homotrimer counterparts. Crosstalk between fusion subunits of Omicron BA.2 and Wuhan-Hu-1 G614 revealed a stabilizing effect of the G614 protomer on the inherently less stable fusion subunit in Omicron. Our findings provide insight into the structural dynamic profiles of co-expressed immunogen display, highlighting important considerations for polyvalent formulations that are being pursued to provide broad protection against highly variable pathogens.

## Introduction

SARS-CoV-2 has been circulating in the human population since 2019, undergoing rapid evolution and diversification under selective pressure from immune responses to infection and vaccination [1,2]. Much of the resulting variation occurs in the viral spike (S) glycoprotein, which mediates entry into host cells by attaching to angiotensin-converting enzyme 2 (ACE2) receptor then carrying out the process of virus-host membrane fusion [3–5]. Understanding the effect of sequence variations in S is important for studies of virus adaptation in response to host immunization and development of effective vaccines.

The D614G mutation was one of the first significant mutations to emerge in S. Located at the interface of receptor-binding (S1) and fusion (S2) subunits, this residue substitution was proposed to increase the stability of S and infectivity of the virus [6]. Structural studies using an engineered pre-fusion S ectodomain construct showed that D614G exhibits enhanced contacts at the interface of S1 and S2 subunits and a bias towards adopting a receptor-binding domain (RBD) “up” configuration compared to the original Wuhan-Hu-1 WT (Hu-1) S [7–13]. Most mutations in S occur in the RBD, leading to increased affinity for human ACE2 and escape from neutralizing antibody (nAb) pressure [14,15]. Indeed most nAbs elicited after natural infections target the RBD on the S1 subunit where they block ACE2 receptor binding [14–16]. Notable hotspot mutations in the RBD, such as N501Y, E484K/A, and K417N/T, combined with D614G mutation, have led to peaks in infection waves associated with variants of concern (VOCs), before major vaccination administrated to the human population was initiated in 2021 [1,17,18].

Both antigenic drift and antigenic shift during SARS-CoV-2’s wide-spread propagation led to improve viral fitness. In late 2021, the Omicron variant emerged, bearing extensive mutations in the RBD and throughout the spike assembly, with enhanced ability to evade immune responses and increased transmissibility [19,20]. Omicron spread rapidly worldwide and diversified into numerous subvariants such as BA.1, BA.2, and BA.5, and their offspring subvariants. XBB, which emerged later, has been suggested to be a recombinant strain following coinfection of a shared host cell by separate strains [21–23]. This recombinant nature allows XBB to have the N-terminal homologous amino acid sequence to variant BJ.1 up to position 459, and the C-terminal sequence from subvariant BM.1. XBB thus inherited structural features from both subvariants that confer enhancements in human ACE2 binding and immune evasion [24,25].

Structure-based vaccine design featuring the display of entire Hu-1 S or RBD antigens have shown protections in preventing severe infections [26–31]. In particular, the efficacy of mRNA vaccines encoding modified S proteins was demonstrated in the COVID-19 pandemic [32,33]. The two most prominent mRNA vaccines developed for COVID-19, Pfizer-BioNTech’s BNT162b2 [26] and Moderna’s mRNA-1273 [29] vaccines, encoding full-length, two-proline-stabilized Hu-1 S (S-2P), demonstrated high efficacy on a global scale by leveraging the human body’s cellular machinery to produce antigens and elicit an immune response [34–36].

The severe pandemic situation and rapid evolution of circulating virus raised the demand for vaccines with high efficacy that could provide broad-spectrum protection against co-existing and newly emerging variants. In response to the emergence of the Omicron VOC’s circulation, a special bivalent mRNA booster vaccine was developed and deployed in fall 2022. This bivalent vaccination contained a 1:1 ratio mix of mRNA-LNPs encoding ancestral Hu-1 and Omicron variants and delivered to human cells with the aim of producing both S antigens to elicit a protective immune response covering both variants [37]. Clinical trials indicated improved protection compared to non-vaccinated and monovalent-vaccinated groups [38]. Previous studies of nanoparticle-based RBD-displaying vaccines suggested the co-display of mixtures of RBDs on multivalent nanoparticles also elicited broader neutralizing antibodies [39].

Relatively little investigation has been reported regarding the effect of co-transfection of host cells with more than one mRNA, with most of the focus being devoted to the downstream immune responses. In the case of bivalent mRNA COVID vaccines, where two mRNA sequences may be delivered to the same cell, in addition to displaying both S antigens on the membrane surface, concurrent translation of mRNAs has the potential to result in translated peptides co-assembling into a mosaic S heterotrimer. To our knowledge it has not been determined whether protomers from different SARS-CoV-2 S constructs can co-assemble as a mosaic heterotrimer and whether co-assembly impacts the trimer’s stability, dynamics and antigenicity.

To address these questions from structural and dynamic perspectives, we investigated the nature of spike antigens produced by co-transfection of cells with different combinations of SARS-CoV-2 variants. We demonstrated assembly of combinatorial mixtures of trimers including homotrimers and mosaic trimers assembled by genetically close strains Omicron BA.2 and XBB (OX), as well as distant strains Hu-1 D614G (G614) and Omicron BA.2 (OG). Hydrogen/deuterium-exchange mass spectrometry (HDX-MS) and biolayer interferometry (BLI) were used to investigate dynamics of the components within the mosaic S assemblies and to determine any impact on RBD structural ordering and receptor-binding site accessibility to receptor and antibodies.

Understanding whether such mosaic heterotrimers can form and their implications on the structural properties of S protein is important, as it could influence the way the antigen presents key epitopes to the host immune system, thus impacting vaccine efficacy. These issues are directly relevant to numerous vaccine studies that are pursuing for example, pan-coronavirus or “universal” influenza vaccines that rely upon co-administration of panels of antigen variants in hopes of eliciting protection against highly variable, rapidly evolving viruses [40,41]. The study we report here provides structural dynamic information to help understand the impact of co-transfection and co-expression on the combinatorial antigens that are produced in these scenarios.

## Results

### Mosaic spike trimers can stably assemble from genetically close as well as distant SARS-CoV-2 variants

**Fig 1:**
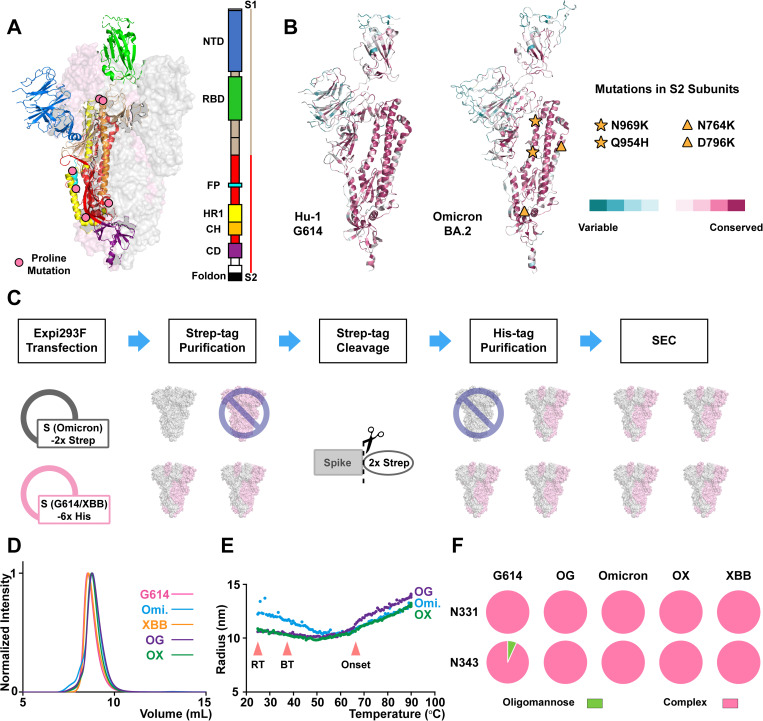
Mosaic S-6P heterotrimer construct sequences, purification process, and characterizations. **(A)** Color-coded S-6P domains on the cartoon diagram with annotated domain organization (PDB: 6VSB). S1: receptor-binding subunit; S2: fusion subunit; NTD: N-terminal domain; RBD: receptor-binding domain; FP: fusion peptide; HR1: heptad repeat 1; CH: central helix; CD: connector domain. **(B)** Structural conservation (ConSurf) maps of Hu-1 G614 (PDB: 6VSB) and Omicron BA.2 (PDB: 7XIW) S**.** Hu-1 G614 and Omicron BA.2 ConSurf maps indicate highly conversed S2 subunit sequences and structures (XBB shares the same S2 subunit sequence as Omicron BA.2). Mutations located in the S2 subunits are indicated with stars (at the trimer interface) and triangles (at the surface). **(C)** Co-expression and tandem columns purification of mosaic heterotrimer. Strep-tag affinity purification removes homotrimer of construct with only His-tag (G614 and XBB). His-tag affinity purification continues removing the homotrimer of Omicron construct with only Strep-tag. **(D)** SEC chromatograms of homotrimers and mosaic heterotrimers purifications. Major peak eluting at ~9 mL reveals the trimeric S integrity. **(E)** Thermal stability measurements on Omicron homotrimer and heterotrimers from 25 °C to 90 °C. RT: room temperature, which HDX experiments are performed under; BT: body or physiological temperature; Onset: onset of the unfolding. **(F)** N-glycosylation profiles on all S constructs indicate predominant complex forms at RBD N331 and N343 sites.

To determine whether co-transfection mimicking bivalent mRNA expression could result in mosaic SARS-CoV-2 S heterotrimer formation, we sought to co-express hexaproline-mutated S ectodomains (S-6P) which were designed to maintain soluble S trimers in the antigenic pre-fusion form [42], from G614, Omicron and XBB S variants (Fig 1A). The ConSurf maps [43] of G614 and Omicron indicate the S2 subunits, especially the central helical domains, are highly conserved (Fig 1B). Total S expression levels were compared with equal amounts of total plasmid DNA transfected under nine conditions: Omicron only, G614 only, XBB only, Omicron-G614 co-transfection (2:1, 1:1, and 1:2), and Omicron-XBB co-transfection (2:1, 1:1, and 1:2). Western blot using an antibody targeting a linear epitope in the S2 subunit that is conserved across all three S variants showed that all conditions expressed similar levels of total S protein in the supernatant (S1 Fig). This indicates that all three S-6P variants have similar transfection efficiency and expression levels under our experimental conditions, with no significant interference from co-transfection of Omicron and G614 variants, and of Omicron and XBB variants.

To evaluate if mosaic heterotrimers can be formed and how they affect trimer stability, we prepared mosaic spike from Omicron-G614 (OG) and Omicron-XBB (OX) combinations side-by-side with control preparations of Omicron, G614, and XBB homotrimers (Figs 1C and S1). Using the optimized purification protocols and low-passage, viable Expi293F cells, the best yields from parallel purifications of all five constructs were obtained (S1 Table). The data agree with the total S expression result and show that the co-expression did not interfere the S trimer production.

Nearly 90% of the protein eluted as a single peak at 8.5–9 mL from the size-exclusion chromatography (SEC) at 4 °C, consistent with well-formed trimers with comparable yields around 4–5 mg (Fig 1D). Both G614 and XBB homotrimers reflect sharp peaks that eluted slightly earlier than Omicron-containing S trimers due to the presence of affinity tags. Omicron homotrimer, eluted later from SEC, showed a modest shoulder eluting slightly before the primary chromatographic peak. The particle hydrodynamic radius and polydispersity measured by dynamic light scattering (DLS) also indicated that Omicron homotrimer had slightly larger radius and polydispersity than other S trimers at 25 °C (S2 Table). Thermal melt experiments monitoring particle radius by DLS provided a means of measuring the stability of S assemblies. Both OG and OX mosaic trimers indicated a smaller radius than Omicron homotrimer under room temperature and physiological temperature ranges (Fig 1E). The trimeric integrity and morphology of mosaic heterotrimers were also confirmed by negative-stain electron microscopy (nsEM) images (S1 Fig). Therefore, both Omicron and XBB protomers, which possess exactly the same S2 subunit sequence, and Omicron and G614 protomers, which carry numerous mutations around the S1/S2 cleavage site and S2 subunit (Fig 1A), could form mosaic heterotrimers with limited alterations in global trimeric structure and morphology.

Furthermore, based on the yield ratios of the isolated mosaic heterotrimer in the elution to the Omicron homotrimer in the flow-through from the last His-tag purification step (S1 Table, OG: 4.7 mg/1.9 mg; OX: 3.6 mg/1.2 mg), the mosaic trimers were estimated to be ~ 60% of the total trimer yield from co-transfection, close to the theoretical percentage for stochastic trimer assemblies from 1:1 co-transfection. This suggests that, in both genetically close and distant strains co-transfection, homotrimer formation did not overwhelm heterotrimer formation, and the presence of heterologous S material did not lead to noticeable misassembly or aggregation.

Another important factor that influences immunogenicity of viral glycoproteins is the glycan profile on the displayed antigens. N-linked glycans can contribute to epitopes that antibodies target [44,45]. Additionally, in SARS-CoV-2 S, N-linked glycans on the RBD have been suggested to affect trimer open/closed state equilibria, which could modulate exposure of some epitopes [46]. To determine how mosaic S heterotrimers glycoprofiles compare with their respective homotrimers, we carried out glycan profiling for the set of mosaic and homotrimer spikes. Our results show that both N-glycosylation sites on the RBD are predominantly complex forms across all five trimeric assemblies (Fig 1F). The glycan profiles we determined in our constructs are consistent across purification batches and in agreement with previous studies on the soluble pre-fusion S ectodomain constructs [47] as well as protein nanoparticle vaccines featuring pre-fusion S or RBD antigens [31,48] that have been shown to elicit potent neutralizing antibody responses. For the rest of the N-glycosylation sites we characterized, the glycan profiles are mostly unaltered in mosaic S trimers except for the N801, which exhibited more processing towards complex forms in OG heterotrimer (S2 Fig).

### Dynamic features of Omicron and XBB protomers in the mosaic heterotrimer are similar to their homotrimer counterparts

Next, we carried out HDX-MS structural dynamic analysis comparing three S-6P homotrimers: G614, Omicron and XBB in order to obtain baseline structural dynamic profiles against which we could compare structural dynamics and trimer integrity profiles of the OX and OG mosaic heterotrimers.

**Fig 2:**
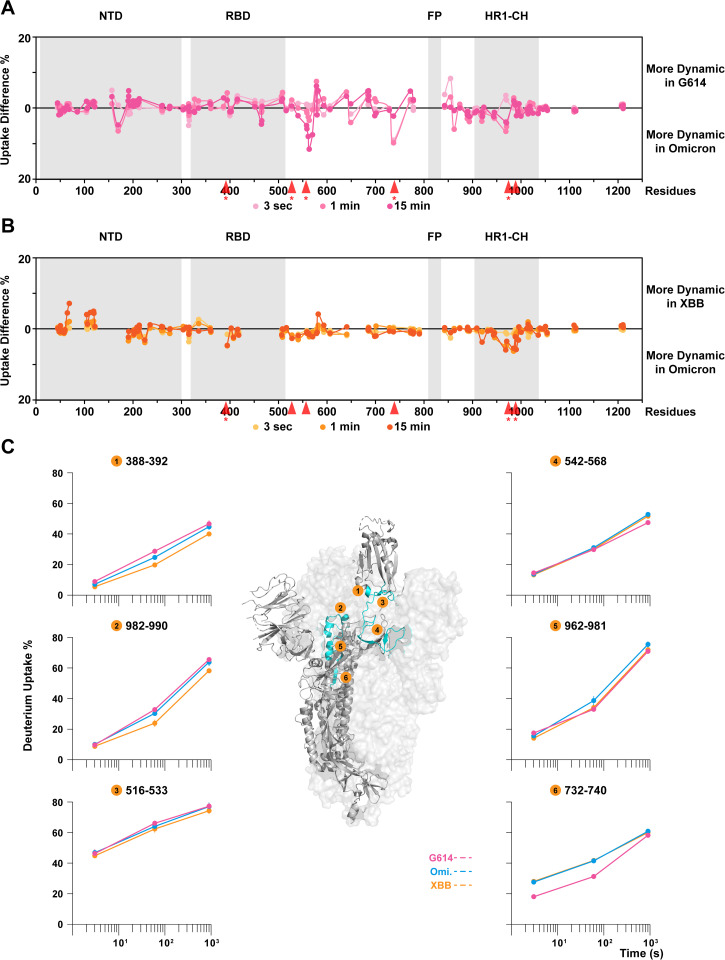
Structural dynamic comparisons between G614, Omicron, and XBB S-6P homotrimers. Differential deuterium uptake plots of **(A)** G614 versus Omicron and **(B)** XBB versus Omicron with **(C)** zoom-in homologous peptide comparisons in the region exhibiting major differences, reveal the strain-specific dynamic profiles. Peptide locations are indicated by red arrows in panel A and B. Asterisk next to the red arrow indicates statistically significant difference between uptake levels on that peptide. Error bars (SD from n > 3) less than 1.5% were masked by the data points. PDB: 6VSB.

HDX-MS analysis on G614, Omicron and XBB homotrimers revealed isolate-specific differences in conformational preferences and S2 dynamics highlighting regions of the polypeptide that were ordered differently among these three homotrimer antigens (Figs 2A, 2B and S3). In our previously reported comparison of S-2P trimers [13], peptides 388–392 and 982–990 were found to serve as effective reporters for RBD up/down configurations as increased dynamics near the C-terminus of the RBD facing the apex of central helices (peptide 388–392) and the central helical apex (peptide 982–990) were observed when S adopts an open conformation. In the present experiments, both of these peptides indicated that dynamics were greater in G614 than in Omicron, which was also significantly greater than in XBB (Fig 2C, peptide #1 and #2). When S is in its open state, the RBD-up conformation also affects dynamics in the S1 hinge region connecting RBD to the rest of the spike trimer. For example, the N-terminal peptide 516–533 connecting to the RBD, showed increased dynamics in response to RBD shifting up, while peptide 542–568, was more protected by the neighboring protomer facing towards the trimer core (Fig 2C, peptide #3 and #4). These dynamic changes all support the conclusion that the G614 S-6P trimer exhibits a greater conformational bias toward populating an open state than Omicron and XBB S-6P trimers.

Sequence variations in the N-terminal domain (NTD) and RBD, especially in the receptor-binding motif (RBM) regions among the three constructs resulted in different digestion patterns, limiting the set of homologous peptides that could provide direct comparisons in these two regions (Fig 2B). By contrast, Omicron and XBB share the same S2 sequences and digestion patterns. HDX-MS analysis revealed that the only significant dynamic differences in the S2 subunit were observed in the heptad repeat 1 (HR1) region reflecting a less open state in XBB (Fig 2C, peptide #2 and #5, and S3 Table). Residue substitutions around the S2’ and HR1 region in Omicron and XBB relative to the ancestral Hu-1 G614 variant yielded differences in S2 subunit dynamics near the S2’ site, where both Omicron and XBB showed increased deuterium uptake over G614 (Fig 2C, peptide #6, and S3 Table). Overall, summarizing the isolate-specific dynamics in G614, Omicron and XBB S homotrimers: G614 S adopted more open conformations with stabilized S2 subunit; Omicron S showed less open conformations than G614 but possessed greater dynamics in the S2 subunit from the HR1 domain extending to the fusion peptide-proximal region (FPPR); and XBB S indicated the least open state preferences and compromised S2 subunit dynamics. The dynamic features in G614 and Omicron S closely agree with previously reported results [49].

To understand whether the formation of mosaic heterotrimers could alter the conformational preference, structural dynamics, and epitope exposure of these antigens, we compared the HDX-MS results from homologous peptides in the mosaic S heterotrimer with those in the corresponding homotrimers. Since the homologous peptides with identical sequence from two constructs will co-elute in HDX-MS, a multi-modal mass envelope is anticipated if a given peptide behaves differently in the different protomers. However, in the cases when the separation was not great enough to deconvolute the bimodal populations into individual protomer contributions, a broadened mass envelope reflecting the average of two underlying populations is observed.

**Fig 3:**
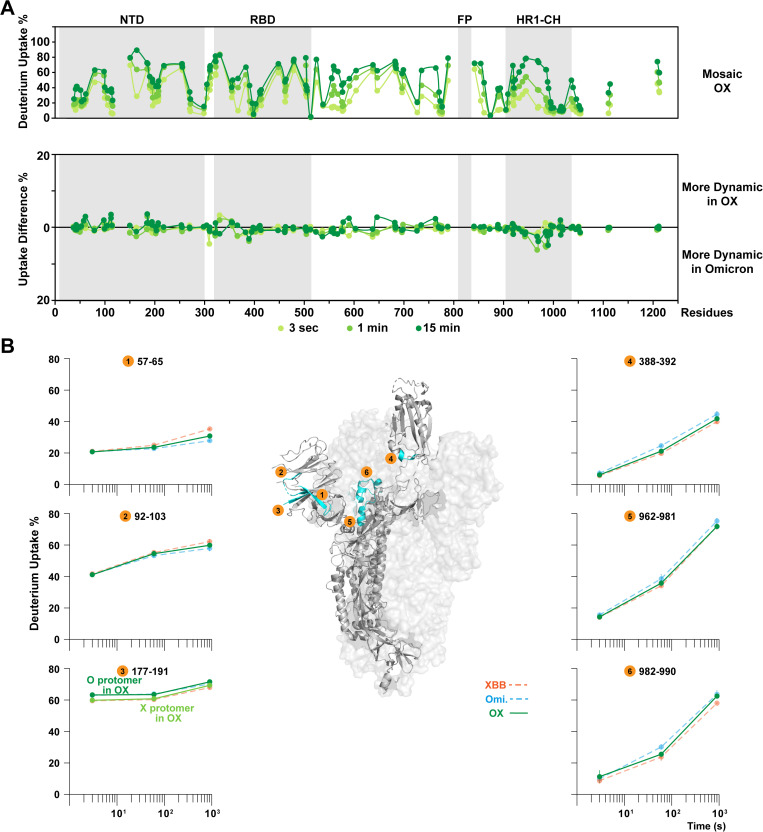
Structural dynamic differences upon forming mosaic Omicron-XBB heterotrimers. **(A)** Deuterium uptake plots and differential plots of Omicron-XBB mosaic heterotrimer and **(B)** pairwise comparisons in homologous peptides, depict dynamic features of mosaic OX heterotrimers. Peptide #1-3 featuring NTD, reveal the NTD dynamics are retained as in homotrimers. In peptide #3, the darker green plot indicates the behavior of Omicron protomer in the OX mosaic trimer while the lighter green plot indicates the behavior of XBB protomer in the OX mosaic trimer. Peptide #4-6 showed the dynamic impacts on spike conformational states, where OX has the intermediate uptake levels. Error bars (SD from n > 3) less than 1.5% were masked by the data points. PDB: 6VSB.

In the Omicron-XBB (OX) mosaic S trimer, the Omicron and XBB protomers mostly retained the dynamic features of their respective homotrimers (Figs 3 and S4). The dynamics of NTD and RBD from each protomer were not significantly affected by mosaic trimer formation as can be seen in the differential plot compared against the baseline Omicron homotrimer (Fig 3A). The peptide uptake profiles in the NTD of OX reflect the average of corresponding uptake levels in Omicron and XBB homotrimers (Fig 3B, peptide #1 and #2).

Homologous peptides such as the peptide spanning residues 177–191 (Fig 3B, peptide #3), where the Q183E substitution in the XBB sequence produced peptides that differ by the single amino acid substitution, could also be distinguished in the mass spectrometer and directly compared with counterparts in the homotrimers. The peptide in the Omicron protomer of the OX mosaic trimer showed the identical dynamic profile as in the Omicron homotrimer and the peptide in XBB protomer matched the XBB homotrimer peptide as well (Fig 3B, peptide #3). These examples thus demonstrated that Omicron and XBB protomers mostly retained the isolate-specific dynamic features over the immunodominant NTD and RBD domains.

Besides the local structural orderings, overall, the dynamic profile of the mosaic OX trimer was found to present as a hybrid between Omicron and XBB (Fig 3). Regions that are structurally associated with S open/closed states such as the RBD C-terminus (Fig 3B, peptide #4) and apex of the central helix (Fig 3B, peptide #5 and #6) exhibited dynamics intermediate between the two homotrimers. The conservation of isolate-specific dynamics in the NTD and RBD as well as conformational preferences indicates relatively little crosstalk and alteration in behavior of the heterologous protomer in the OX co-assembled spikes, most likely due to the commonality of their S2 subunits.

### A greater degree of crosstalk is observed between protomers in the Omicron-G614 mosaic heterotrimers

**Fig 4:**
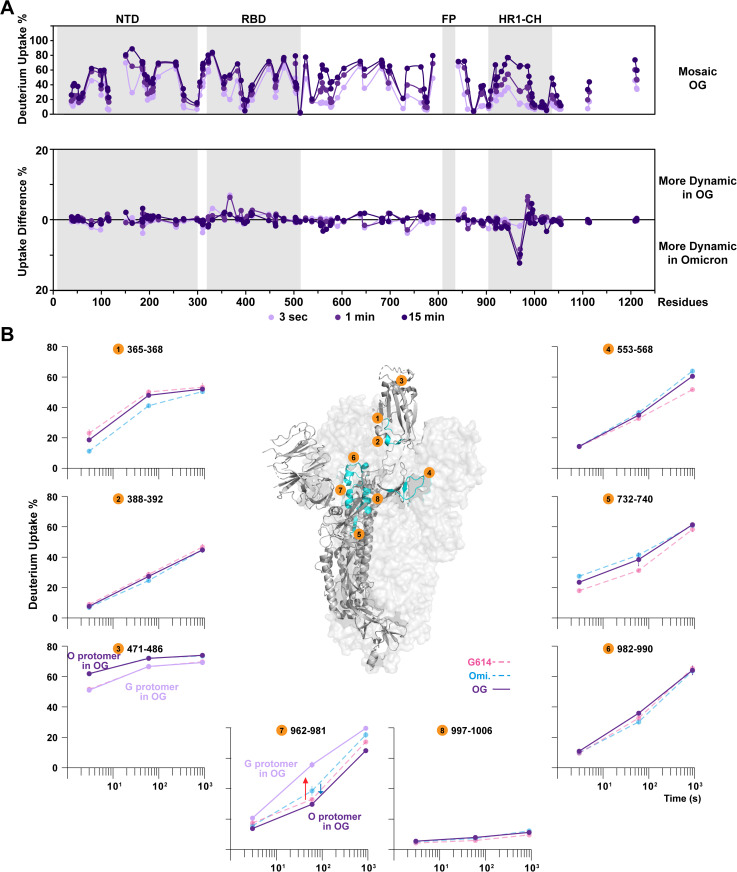
Structural dynamic differences upon forming mosaic Omicron-G614 heterotrimers. **(A)** Deuterium uptake plots and differential plots of Omicron-G614 mosaic heterotrimer and **(B)** pairwise comparisons in homologous peptides, depict dynamic features of mosaic OG heterotrimers. Peptide #1-3 featuring RBD, reveal the RBD dynamics are retained as in homotrimer. Peptide #4 is on the S1 hinge region, interacting with neighboring NTD, shows similar dynamic profile to Omicron. Peptide #5-7 indicate the increasing dynamics over FPPR in the OG heterotrimer. In peptide #3 and #7, the darker purple plot indicates the behavior of Omicron protomer in the OG mosaic trimer while the lighter purple plot indicates the behavior of G614 protomer in the OG mosaic trimer. Peptide #8 confirms the spike integrity reflected by the well-protected central helix core. Error bars (SD from n > 3) less than 1.5% were masked by the data points. PDB: 6VSB.

We next investigated the dynamic behavior in the Omicron-G614 (OG) mosaic heterotrimer. This combination enabled us to examine whether a mosaic S trimer with divergent S2 subunits may exhibit more prominent effects on inter-protomer packing or allosteric effects across the trimeric spike assembly that could affect their antigenicity. In the OG mosaic trimer, similar effects were observed in the RBD as in the OX case, which exhibited uptake levels intermediate between those in Omicron and G614 homotrimers (Fig 4B, peptide #1 and #2, and S5 Fig). Peptide 471–486, spanning the loop connecting the RBD core and the RBM located at the surface of RBD, is a target for neutralizing antibodies and is highly mutated during viral evolution, conferring immune escape [50,51]. The S477N, T478K, and E484A substitutions in peptide 471–486 observed in Omicron versus G614 protomers enable the homologous peptides to be distinguished, allowing direct comparison between the behavior of this peptide in the two types of protomers. In this case, the uptake level in each protomer matched the behavior seen in the respective homotrimer (Fig 4B, peptide #3). Notably, in contrast to the conformational preferences observed in the OX heterotrimer, the OG heterotrimer appeared to favor open conformations. Specifically, the peptides reporting on RBD configurations in the OG heterotrimer more closely paralleled their behavior in the G614 homotrimer, showing the dominant influence of the G614 protomer on the mosaic OG assembly (Fig 4B, peptide #1, #2 and #6, and S5 Fig). One of the exceptions to this G614 dominance was observed in the hinge region (Fig 4B, peptide #4), where the dynamic behavior of this peptide in OG heterotrimer more closely recapitulated its behavior in the Omicron homotrimer. While increased dynamics at C-terminal RBD (peptide #2) and helical apex (peptide #6) were consistent with greater exposure in an open conformation, the dynamics of the peptide 553–568 loop, which is located at the interface between RBD and a neighboring NTD, was found to be more affected by the positioning and packing of the NTD on the neighboring protomer (Fig 4B, peptide #4). The dynamic features at this site in the OG heterotrimer mirrored the behavior in the Omicron homotrimer indicating that the NTD maintained a similar position and conformation as in Omicron homotrimer, despite the RBD favoring the more open state in the same OG mosaic trimer. In the mosaic trimer, these structural motifs appear to be somewhat decoupled in their dynamic behavior.

**Fig 5:**
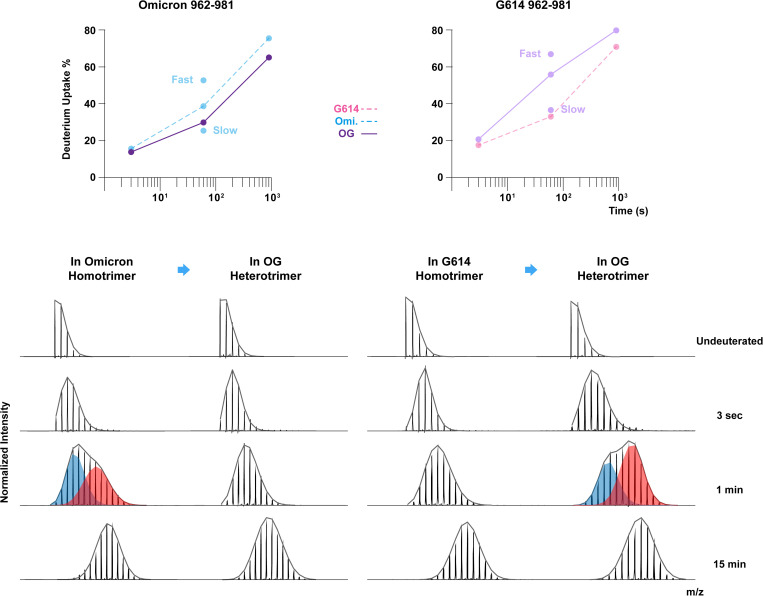
Protomer-specific dynamics near S2 subunit apex in the Omicron-G614 heterotrimers. Peptide 962-981 containing a N969K mutation in Omicron construct is used to differentiate deuterium uptake profiles between Omicron and G614 protomers. Dynamic changes in Omicron protomers (left panel) and G614 protomers (right panel) from their homotrimers to OG heterotrimer are compared. Fast and slow uptake levels labeled on the plots, correspond to red (fast) and blue (slow) populations of the bimodal mass envelopes at 1-minute time point.

Prominent dynamic changes in the OG heterotrimer were found in the FPPR region between S1/S2 and S2’ cleavage sites (Fig 4B, peptide #5) and HR1 domain (Fig 4B, peptide #7). Notably, homologous peptides in the region 962–981 were differentiated from two protomers due to the N969K mutation in Omicron. This helix-loop-helix peptide was intrinsically more dynamic in the Omicron homotrimer than the G614 homotrimer, in agreement with a previous study [49], but the trend was reversed in the Omicron protomer and the G614 protomer when they co-assembled (Fig 4B, peptide #7). Examining the mass spectra revealed a bimodal mass envelope for this FPPR peptide in the Omicron homotrimer at the 1-minute time point (Fig 5) with a fast-uptake population in the Omicron homotrimer that could adopt a more exposed, dynamic conformation. However, the same region in the Omicron protomer in the mosaic OG trimer showed decreased uptake levels with less detectable peak broadening and suppressed bimodal spectra which mostly resembled the slow-uptake population in the Omicron homotrimer (Fig 5). This suggests that the FPPR in the Omicron protomer of the OG mosaic trimer stably maintains its ordered conformation. On the other hand, the homologous peptide in the G614 protomer showed the opposite trend, exhibiting significantly increased dynamics, in the OG mosaic trimer (Fig 5). Enhanced dynamics in the G614 protomer of the OG mosaic trimer was reflected by the presence of novel dynamic conformations in FPPR, that gave rise to a bimodal mass envelope at the 1-minute time point compared to the unimodal ordered conformation in G614 homotrimer (Fig 5). This phenomenon indicates that Omicron-specific mutations Q954H and N969K can allosterically induce the neighboring protomer to adopt highly dynamic conformations in the FPPR.

The crosstalk between protomers in the OG mosaic heterotrimer gave rise to novel structural and dynamic features that S homotrimers did not present. Specifically, the mosaic heterotrimer favored an open state as seen in the G614 homotrimer, with the isolate-specific orderings in NTDs and RBDs for the Omicron protomers mostly retained. The pre-fusion S helix-loop-helix region at FPPR became more ordered in the Omicron protomers with the induction of highly dynamic conformation in the G614 protomer over the same region. Despite such prominent dynamic effects in the S2 subunit, the OG mosaic trimer integrity and stability were well-maintained, as indicated by constantly protected central helical domains from HDX-MS (Fig 4B, peptide #8, and S5 Fig), thermal stability results (Fig 1E), and nsEM images (S1 Fig). Thus, the sequence differences in S2 between the ancestral G614 subvariant and prevalent Omicron subvariant were not sufficient to impact the trimer’s ability to assemble but did result in modulation of the trimer’s dynamic profile at positions across the spike assembly.

### Antigenic effects of mosaic trimer formation

Previous studies have reported that over 90% of neutralizing antibodies targeted RBD and NTD [14–16]. Retaining the structural ordering and functioning in NTD and RBD is important for assessing the antigenicity as well as vaccine efficacy. To further investigate whether the localized structural dynamic differences in the mosaic trimers have antigenic effects, bio-layer interferometry (BLI) was used to compare human ACE2 (hACE2) and antibody binding kinetics for mosaic versus homotrimer spikes.

**Fig 6:**
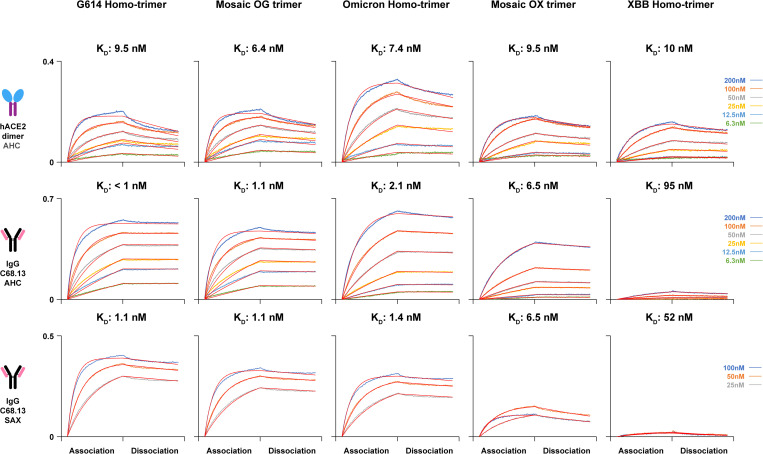
RBD structural orders of mosaic S-6P heterotrimers. BLI measures K_D_ on five spike trimers interacting with dimeric human ACE2 and monoclonal antibody C68.13. AHC: Anti-human IgG Fc Capture biosensors used to load and immobilize Fc-hACE2 dimer and C68.13 IgG. SAX: Streptavidin biosensors used to load and immobilize biotinylated S-6P trimers for detecting soluble C68.13 IgG. Binding curves are aligned at the start of association step and fitted globally with a 1:1 ligand binding model (red fitting curves).

In the dimeric hACE2 binding experiments, anti-human IgG Fc capture (AHC) immobilized Fc-hACE2 strongly bound all five constructs with K_D_ < 10 nM (Fig 6), in agreement with previously reported K_D_ values measured by surface plasmon resonance [52]. We then tested binding with monoclonal antibody (mAb) C68.13 that competes with ACE2 binding and has been reported to exhibit 2–3 orders of magnitude weaker inhibition of XBB than G614 and Omicron [53]. This allows us to selectively examine how the critical RBD epitope presentation is modulated on the mosaic heterotrimer. In our binding assays using AHC biosensors, the G614 homotrimer, Omicron homotrimer and OG mosaic trimer were found to bind C68.13 with high affinity, K_D_ ~ 1 nM (Fig 6). Flat dissociation curves resulted in k_off_ rates in lower 10^-4^ levels, close to the detection limit, indicating very stable binding. The XBB homotrimer bound C68.13 with K_D_ ~ 95 nM, 50-to-100-fold weaker binding to this mAb than G614 and Omicron homotrimers (Fig 6). To test whether the BLI configurations with immobilized IgG and S analyte were contributing to the measured binding affinities, we also performed binding assays using Streptavidin biosensors, which enabled biotinylated S constructs to be captured. Binding to soluble C68.13 yielded the similar binding behaviors and affinities. The G614 homotrimer, Omicron homotrimer and OG mosaic trimer showed C68.13 binding affinity ~1 nM, while XBB homotrimer showed 50-fold weaker binding and OX mosaic trimer showed a moderately compromised K_D_ of 6.5 nM (Fig 6). Compared with the immobilized bivalent IgG, immobilized S trimers for IgG detections indicate both higher k_on_ and k_off_ values but the resulting K_D_ closely matched the measurements with immobilized IgG (S4 Table). These data indicate that the mode of interaction between the RBDs and antibodies was relatively unaltered by the presence of adjacent protomers with weak binding affinities.

Compared to the Class 2/3 RBD mAb C68.13, Class 3 mAb C68.61 binds to a more conserved epitope toward the RBD core regions [53]. C68.61 showed strong binding to all five constructs in both BLI configurations with immobilized IgG and with immobilized biotinylated S (S6 Fig). Slow dissociation occurred for both IgG and Fab forms of C68.61 in these binding assays (S4 Table). These results indicate that the high affinity binding of these RBD-targeting C68 mAbs, is not primarily due to avidity or bivalent binding.

In addition, we performed competitive binding assays with C68.13 and hACE2. Omicron, OX and XBB S trimers were first bound with excessive C68.13 IgG to saturate RBDs on the Omicron protomers. When hACE2 was subsequently introduced, it was able to bind to the unoccupied RBDs on the XBB protomers, resulting in a secondary BLI response. The OX mosaic heterotrimer exhibited an intermediate response between Omicron and XBB homotrimers, indicating the accessibility and binding were less affected by the presence of the adjacent heterotypic protomer (S7 Fig). Our BLI results align with the inferences from the HDX experiments, suggesting that mosaic heterotrimers (OX and OG) impose limited restriction on RBD dynamics and receptor/antibodies binding.

## Discussion

Glycoproteins on enveloped viruses such as influenza, HIV, and coronavirus are the primary antigenic features on the virus surfaces. The stability, dynamics, and structural ordering of these multimeric assemblies influence the display of antigen epitopes and their recognition by neutralizing antibodies. Viruses with RNA genomes including SARS-CoV-2 evolve rapidly when spreading through large populations and adapting to escape host immune responses. In order to combat emergence of such viral diversity and maintain or increase breadth of immune responses, many strategies under investigation combine antigens from multiple variants and viral strains into polyvalent antigen cocktails. In the case of nucleic acid-launched vaccines such as those employing mRNA, immunization with mixtures of genes encoding the antigens from various strains are delivered to the vaccinee such that co-transfection of cells has the potential to lead to co-expression of two or more antigen variants. As we have shown here, this also can lead to co-assembly of mosaic antigens. Such a strategy of combining a bivalent antigen cocktail was used for a COVID-19 booster immunization, deployed in 2022, which combined proline-stabilized, pre-fusion Hu-1 and Omicron variants [37]. Of note, such a situation of co-expression and co-assembly of antigen variants may also take place if a cell is co-infected with more than one strain of virus during superinfection of the host. Evidence of coinfection for coronaviruses is abundant, both involving SARS-CoV-2 variants, which has led to genetic recombinants such as XBB [54], as well as even more divergent CoV species that may coinfect a host organism [55]. When the two coinfecting variants produce S proteins, co-assembly into mosaic spikes is a possible outcome. The general conservation of core S2 motifs such as the central helical domain, would facilitate co-assembly. Progeny virions displaying mosaic trimers, could in theory confound neutralization if one variant is resistant to existing sera, resulting in a combinatorial phenotype. However, since each particle would likely package a single variant’s genome (assuming a single copy is packaged per particle) [56], subsequent rounds of infection may not lead to persistent production of mosaic antigens, unless new rounds of cell coinfection occur. Such a situation likewise may emerge in viruses such as HIV-1, where a chronically infected individual is exposed to a second distinct HIV-1 strain leading to superinfection of the host [57]. Beyond co- and super-infection, rapidly evolving RNA viruses can generate quasispecies swarms of related genotypes resulting from spontaneous mutation. The impact of co-infection with such genotypic diversity on co-assembling antigen structure to our knowledge has not been explored but potentially could be investigated using approaches such as deep mutational scanning to produce large pools of mutants resembling a viral quasispecies [58].

The three SARS-CoV-2 S variants examined here: Hu-1 G614, Omicron BA.2, and XBB, were selected based on their genetic distances and significance as vaccine immunogens from distinct waves of the pandemic. The G614 variant, close to the ancestral Wuhan-Hu-1 WT and first detected in early 2020, led to subsequent infection waves worldwide before major vaccination was deployed [6]. In contrast, Omicron subvariants and XBB emerged in late 2021 and 2022, respectively, carry mutations that emerged after the administration of two or three vaccine doses in most developed countries and widespread natural infection worldwide [19,21]. The genetic distance between G614 and Omicron is relatively large compared to the distance between Omicron and XBB. In particular, XBB is a recombinant variant formed from two Omicron subvariants, resulting in notably different S1 but identical S2 subunit sequences compared to Omicron BA.2. Our choice of S variants thus enabled us to investigate the impacts of the bivalent mRNA vaccine-expressed antigens as well as the structural and dynamic impact of S2 subunit sequence similarity and differences on mosaic antigen display.

We found that mosaic S trimers from co-transfection maintained the overall protein integrity and morphology relative to the corresponding homotrimers. The yield of mosaic trimer closely matched the theoretical yield for respective homotrimers, suggesting a negligible cost for co-expressing and co-assembling protomers from different variants into mosaic trimers. We speculate that this well-ordered S morphology is due to the highly similar S2 subunit sequence and conformation. S2 subunit is also highly conversed across S variants, especially the HR1 and central helix domain (Fig 1B). A trial co-expression with SARS-CoV-2 S and S from another **Sarbeco*virus*, SARS-CoV, sharing less than 80% sequence similarity to SARS-CoV-2 S [3], also resulted in mosaic heterotrimer co-assembly (S1 Fig). Notably, while SARS-CoV S shares 90% S2 subunit similarity, less than the similarities within SARS-CoV-2 variants (> 98%), it possesses the same central helical domain sequence (residues 970–1070) as the SARS-CoV-2 G614 S, which is largely responsible for the ability of these divergent S to co-assemble. We also note that the use of hexaproline-stabilized S ectodomain may suppress variant-specific effects impacting trimer co-assembly, such as the different intrinsic propensities to convert to a post-fusion form and conformational switching [59,60]. The incorporation of proline mutations improved overall purification yields and experimental consistency. We anticipate that the observed effects we describe may be even more prominent for less stabilized diproline, S-2P, trimers that are used in the approved COVID vaccines.

The mosaic heterotrimers we expressed should exist as a mixture of AAB and ABB heterotrimer forms (A and B represent two different protomers) resulting from the 1:1 ratio of transfected constructs. Western blots showed a roughly 1:1 ratio of each heterotrimer form (S1 Fig). Therefore, the deuterium uptake level from HDX-MS reflected a compounded dynamic profile of both AAB and ABB forms. Our conclusion of mosaic heterotrimer dynamics thus likely underestimates specific effects as we could not differentiate between protomer behaviors in AAB- or ABB-specific individually.

Numerous on-going investigations seek to generate immune responses that can provide broad protection against rapidly evolving viruses. Such efforts on pan-coronavirus or universal influenza vaccine studies seek to elicit broad responses by vaccinating with cocktails of antigen variants in some cases co-formulated and in others co-displayed on multivalent nanoparticle platforms [39,61–64]. One hope of such polyvalent display of antigenic variants is to select for antibodies that are able to recognize the conserved features while tolerating variability in other regions through avid binding of multivalent nanoparticles to B-cell receptors. In the case of the mosaic trimers we have analyzed, it may similarly be possible for hetero-protomers in a given co-assembled trimer to engage with B-cell receptors that can bivalently bind to conserved epitope features displayed in two distinct contexts, helping to favor a broad response.

Our experiments have shown that, at least in the SARS-CoV-2 spike system, bivalent co-formulation and transfection of S variants lead to the formation of mosaic heterotrimers that retain structural integrity while exhibiting novel dynamic behaviors distinct from the parental homotrimers. Our experiments demonstrated interprotomer crosstalk especially in mosaic spike heterotrimers from relatively divergent sequences. For future vaccine designs it may be necessary to take into account the potential formation of mosaic heterotrimers and resulting structural and immunological implications following polyvalent nucleic acid vaccination such as for pan-coronavirus and universal influenza vaccines or in targeting highly variable viruses such as HIV.

## Materials and methods

### Plasmid construction

The gene sequences encoding SARS-CoV-2 S ectodomain with hexaproline mutations (S-6P: F817P, A892P, A899P, A942P, K986P and V987P) [42], GSAS substitution at S1/S2 site (RRAR) and C-terminal T4 fibritin foldon were cloned into pCMV plasmid. The open reading frame (ORF) sequence of Hu-1 G614 S-6P construct is the reference Wuhan-Hu-1 sequence (residues 14–1211) with the D614G mutation, C-terminal His-tag and the alterations mentioned above. Omicron BA.2 construct was assembled from the ORF in the pαH Omicron BA.2 S-2P plasmid (Addgene plasmid #184829) into pCMV plasmid and mutated with four additional prolines using HiFi DNA Assembly (NEB). XBB construct was assembled from the ORF in the pVRC8400 XBB S-2P plasmid (Addgene plasmid #160474) into pCMV plasmid and mutated with four additional prolines and a C-terminal His-tag (NEB). The ORF sequence of SARS-CoV (SARS1) S-6P (Tor2 strain [65] with F799P, A874P, A881P, S924P, K968P and V969P) was cloned into pCMV plasmid with the same vector backbone as G614 and XBB construct.

### Transient transfection

293T adherent cell line was used for optimizing co-transfection condition. Cells were split into 12-well plate (1 mL per well) at seeding density (0.1 × 10^6^ cells per well). Confluent cells (0.5 × 10^6^ cells per well) were transfected with total 2 μg DNA and 2 μL Lipofectamine 3000 (Invitrogen) based on Lipofectamine 3000 reagent protocol. DNA being transfected or co-transfected into the cell cultures under nine conditions: Omicron only, G614 only, XBB only, Omicron-G614 co-transfection (2:1, 1:1, and 1:2), and Omicron-XBB co-transfection (2:1, 1:1, and 1:2). After 2 days in the 37 °C incubator (5% CO_2_), 200 μL supernatant from each well was collected for SDS-PAGE to check expression level.

Suspension Expi293F cell culture was used for spike protein expression. For a 700 mL cell culture at confluency (3 × 10^6^ cells/mL), 0.7 mg total DNA (1:1 for co-transfection) and 2.1 mL ExpiFectamine 293 reagent were mixed in Gibco Opti-MEM and added into the cell culture following ExpiFectamine 293 user guide. The transfected culture was incubated at 37 °C and 5% CO_2_ with constantly shaking at 125 RPM for 5 days before harvest.

### Protein purification

On the fifth day post-transfection, Expi293F cells were harvested by centrifugation at 2000 RCF for 30 minutes. Supernatant was vacuum-filtered through 0.45 μm αPES filters and supplemented with Tris-HCl (pH 8.0) and NaCl.

For G614 and XBB spike homotrimers purification, imidazole was added to the supernatant to reach a final concentration of 50 mM Tris-HCl (pH 8.0), 200 mM NaCl and 20 mM imidazole, same as the Ni-IMAC binding buffer composition. A 2 mL Ni-IMAC resin (Thermo Fisher Scientific) was washed and equilibrated with binding buffer and packed for a gravity flow column. Supernatant was loaded and flowed through this gravity flow column, followed by four times 10 mL (5 CV) wash with binding buffer. The bound protein was then eluted from the column with three times 6 mL (3 CV) Ni-IMAC elution buffer (50 mM Tris-HCl, pH 8.0, 200 mM NaCl, 300 mM imidazole). Elution fractions were SDS-PAGE checked, and then combined. Combined spike protein solution was buffer-exchanged to reduce the imidazole level below 1 mM and concentrated to ~1 mg/mL with an Amicon Ultra-15-mL-centrifugal filter (30 K, Millipore), followed by flash frozen in liquid nitrogen and stored in -80 °C freezer.

For Omicron, mosaic Omicron-G614, mosaic Omicron-XBB and mosaic Omicron-SARS1 trimers purification (Fig 1C), ethylenediaminetetraacetic acid (EDTA) was added to reach a final concentration of 100 mM Tris-HCl (pH 8.0), 150 mM NaCl and 1 mM EDTA, same as the Strep-Tactin XT wash buffer composition. A 3.5 mL Strep-Tactin XT 4Flow high-capacity resin was washed and equilibrated with wash buffer and packed for a gravity flow column. Supernatant was loaded and flowed through this gravity flow column, followed by four times 8.5 mL (2.5 CV) wash. The bound protein was then eluted from the column with three times 5 mL (1.5 CV) Strep-Tactin XT elution buffer (100 mM Tris-HCl, pH 8.0, 150 mM NaCl, 1 mM EDTA, 50 mM biotin). Elution fractions were combined, buffer-exchanged to reduce the biotin level below 1 mM and concentrated to a final volume below 1 mL. HRV 3C protease (Pierce) was added accordingly based on the protein solution volume for the digestion to remove TwinStrep tag overnight at 37 °C. On the next day, digested samples were spun at 15000 RCF for 10 minutes to remove possible protein aggregation pellet. The Omicron homotrimer protein could now be flash frozen in liquid nitrogen and stored in -80 °C freezer. For mosaic heterotrimer purifications, samples were flowed through a 1 mL pre-equilibrated prepacked StrepTrap XT column (Cytiva) at 1 mL/min to separate undigested protein. Flow-through was reloaded to the column for better purification results. The final flow-through containing heterotrimer with TwinStrep tag removed was collected and buffer-exchanged to the nickel column binding buffer. Samples were flowed through a 1 mL pre-equilibrated prepacked HisTrap HP column (Cytiva) at 1 mL/min, followed by 7 mL (7 CV) wash. The bound protein was then eluted from the column with 3 mL (3 CV) elution buffer. SDS-PAGE checked elution fractions were combined, buffer-exchanged to reduce the imidazole level below 1 mM and concentrated to ~1 mg/mL with an Amicon Ultra-15-mL-centrifugal filter (30 K, Millipore). The duo-affinity-column purified mosaic spike heterotrimers were then flash frozen in liquid nitrogen and stored in -80 °C freezer.

All the spike trimers were finally size-exclusion chromatography (SEC) purified before major experiments. 500 μL sample was thawed and spun at 15000 RCF for 10 minutes to remove possible protein aggregation pellet before injecting to Superdex 200 column (GE) on an AKTA Pure system (GE). With the SEC running buffer (10 mM HEPES, 200 mM NaCl, 0.02% NaN_3_, pH 7.5) and flow rate at 0.5 mL/min, spike trimer was eluted at around 8.5–9 mL. The SEC purified spike trimers were concentrated to desired concentrations according to the experiment need.

### Dynamic light scattering

Dynamic light scattering measurements were performed on a DynaPro NanoStar II (Wyatt) to characterize spike trimer integrity, homogeneity and thermal stability. SEC purified spike samples at 0.5 mg/mL were first spun down at 15000 RCF for 20 minutes to remove aggregation pellet. 5 μL of the sample was then injected into a 2-μL quartz cuvette (Wyatt). Each DLS run was measured with twenty 10-second acquisitions at 25 °C by LASER auto-attenuation. Thermal melt measurements were performed from 25 °C to 90 °C with the rate at 0.5 °C/min. Five measurements were taken and averaged at each temperature point.

### Hydrogen/Deuterium-exchange mass spectrometry

For the hydrogen/deuterium-exchange (HDX) experiments, 10 μg each of Hu-1 G614, Omicron BA.2, XBB, mosaic Omicron-G614 and mosaic Omicron-XBB spike trimer was incubated in the deuteration buffer (10 mM HEPES, pH^*^ 7.5, 85% D_2_O, Cambridge Isotope Laboratories, Inc.) at 22 °C for three different timepoints: 3, 60, 900 seconds (five replicates for 3 seconds and four replicates for 60 and 900 seconds). All exchanged samples were immediately mixed with an equal volume of ice-chilled quench buffer containing 8 M urea, 200 mM tris(2-chloroethyl) phosphate (TCEP) and 0.2% formic acid (FA) to pH 2.5, transferred into an autosampler vial with metal cap and flash frozen in liquid ethanol. Samples were loaded to the LC-MS system by an automating platform and analyzed by a Synapt G2 mass spectrometer (Waters) with the settings described in previous publication [66]. Samples were in-line digested into peptides by an immobilized pepsin column (2.1 × 50 mm) and loaded onto CSH C18 trap cartridge (Waters) with loading buffer (2% acetonitrile (ACN), 0.1% trifluoroacetic acid (TFA)) at 800 μL/min. Peptides were then separated by a UPLC CSH C18 column (1.0 × 50 mm, 1.7 μL, Waters) with a 15-min linear gradient from 3% to 40% buffer B (buffer A: 2% ACN, 0.1% FA, 0.025% TFA; buffer B: 99.9% ACN, 0.1% FA) at a 40 μL/min flow rate.

Samples were also run on a Orbitrap Ascend Tribrid mass spectrometer (Thermo Fisher) to obtain MS/MS information for both determining N-glycosylation forms and identifying pepsin digested peptides by Byonic and Byos (Protein Metrics). Peptide mass spectra were further confirmed on DriftScope (Waters) and identified with specific retention time and drift time. HDExaminer (Sierra Analytics) was used to parse the mass spectrometer datasets and calculate deuterium uptake of each peptide identified by the specific retention time and drift time.

### SDS-PAGE and western blot

For all the SDS-PAGE gels using Coomassie Blue staining, 11 μL spike samples were mixed with 1 μL DTT (0.5M) and 4 μL 4 × NuPAGE LDS loading dye (Invitrogen) before loading to 4–12% NuPAGE Bis-Tris gel (Invitrogen) wells. SDS-PAGE gels were run at 120 Volts for 10 minutes and 150 Volts for 60 minutes in MES SDS running buffer (Invitrogen), followed by gel collection and PageBlue (Thermo Scientific) staining.

For SDS-PAGE gels proceeding to western blots, 5 μL spike samples were mixed with 1 μL DTT (0.5M) and 2 μL 4 × loading dye, loaded to the SDS-PAGE gel wells, and run at 120 Volts for 80 minutes. The peptides on the gel were then transferred to the Immobilon-FL PVDF membrane (Millipore) using 10% methanol supplemented NuPAGE transfer buffer (Invitrogen) at 30 Volts for 2 hours. All membranes were blocked in 5% non-fat milk overnight, followed by incubation in primary antibody, respectively, in room temperature for 1 hour. Primary antibody against spike S2 subunit (1A9, mouse, mAb, Invitrogen, MA5-35946) was used in 1:2000; against Strep-tag (mouse, mAb, GenScript, A01732S) was used in 1:2000; against His-tag (mouse, mAb, Invitrogen) was used in 1:2000; against SARS-CoV spike S1 subunit (mouse, mAb, Sino Biological, 40150-MM10) was used in 1:2000 tris-buffered saline-Tween (TBST) solution. Secondary antibody anti-mouse IgG was used in 1:5000 5% non-fat milk blocking buffer in room temperature for 1 hour. The membranes were imaged on the Odyssey M-XS imaging system (LI-COR Biosciences) using 700 nm channel and optimized exposure intensity.

### Bio-layer interferometry

Bio-layer interferometry was used to measure binding kinetics between spikes and hACE2 receptor or neutralizing antibodies. Octet anti-human Fc capture (AHC) biosensors (Sartorius) were pre-incubated in assay binding buffer (10 mM phosphate-buffered saline (PBS), pH 7.4, 0.1% bovine serum albumin (BSA), 0.02% Tween 20) for at least 15 minutes. Spike samples were 2-fold serial diluted with binding buffer from 200 nM to 3.1 nM. Ligand samples were also prepared with binding buffer at 2 μg/mL concentration for dimeric hACE2-Fc and 1 μg/mL for antibodies. Octet Streptavidin (SAX) biosensors (Sartorius) were used to immobilize 15 μg/mL biotinylated spike for antibody detection. In this assay setup, C68 IgGs were 2-fold serial diluted with binding buffer from 100 nM to 25 nM and Fab was 2-fold serial diluted from 200 nM to 50 nM. The binding assay was performed on an Octet BLI system (Sartorius) with 60-second ligand loading, 180-second association and 180-second dissociation processes. The competitive binding assay was performed with 60-second ligand loading, 360-second association with 100 nM C68.13 IgG solution, followed by another 360-second association with 100 nM C68.13 and 100 nM hACE2-Fc solution. BLI kinetics data were processed by ForteBio Data Analysis software (v11.0) with 1:1 binding model fitting.

### Negative-stain electron microscopy

SEC purified spike trimers were diluted to 20 ng/μL for negative-stain electron microscopy (nsEM). Carbon film 300 mesh copper grids (Electron Microscopy Sciences) were glow discharged for 30 seconds in a vacuum chamber. 3 μL of each diluted spike sample was added onto the grid (carbon side), incubated for 1 minute before blotting off with a piece of folded filter paper. The grid was then incubated in one drop of nano-W (Methylamine Tungstate, Nanoprobes) for 1 minute, followed by blotting off excess stain solution to leave a thin layer. The air-dried grid was saved in the grid box and imaged on a FEI Tecnai G2 Spirit TEM (120 kV) with a Gatan Ultrascan 4000 CCD detector. Data collection was performed using Leginon with an image pixel size of 1.6 Å.

### Data analysis

Statistical analysis was done for pairwise comparison between HDX-MS deuterium uptake levels. Technical replicates (n = 5 for 3-second, n = 4 for 1-minute and 15-minute time points) were prepared and run for all five constructs during HDX-MS. Two-tailed t-test was used to report the statistics. All uptake plots include error bars indicating the standard derivations from (n > 3) replicates. Error bars less than 1.5% were masked by the plotted data points on the plots.

Protein structures including ConSurf maps and HDX heatmaps were visualized and exported using PyMOL 3.0 (Schrödinger). Figures were prepared in Adobe Illustrator (v29.2.1).

## Supporting information

S1 Fig Mosaic S-6P heterotrimer purification. **(A)** SDS-PAGE gels with Coomassie blue staining from affinity purification of each S trimer. FT: Flow-through; Elu.: Elution **(B)** Western blot with anti-His primary antibody indicates the efficiency of HRV 3C protease digestion and tandem-affinity purifications in separating mosaic heterotrimers from homotrimers. **(C)** Western blot characterization and quantification of homotrimers and heterotrimers using anti-S2-subunit and anti-His primary antibodies. **(D)** Western blot quantification of the total S expression from different co-transfection ratios. **(E)** Negative-stain electron microscopy images illustrate the morphology of Omicron-G614 heterotrimer and Omicron-XBB heterotrimer. **(F)** SDS-PAGE gel with Coomassie blue staining from affinity purification of Omicron-SARS-CoV (O-SARS1) S trimer. **(G)** Western blot characterization of homotrimers and heterotrimers using anti-S2-subunit and anti-SARS-CoV primary antibodies. O1: O-SARS1 mosaic heterotrimer. This figure is related to Fig 1.
(TIF)

S2 Fig N-glycosylation profiling on S-6P trimers. 17 out of 22 N-glycosylation sites are characterized and compared. This figure is related to Fig 1F.
(TIF)

S3 Fig Dynamic comparisons among G614, Omicron and XBB S-6P homotrimers. Butterfly deuterium uptake plots depict strain-specific S dynamic profiles with highlights in the functionally important domains. This figure is related to Fig 2.
(TIF)

S4 Fig Deuterium uptake plots of mosaic Omicron-XBB S-6P. This figure is related to Fig 3.
(TIF)

S5 Fig Deuterium uptake plots of mosaic Omicron-G614 S-6P. This figure is related to Fig 4.
(TIF)

S6 Fig RBD structural orders of mosaic S-6P heterotrimers. BLI measurements on five biotinylated S-6P trimers interacting with C68.61 IgG and C68.61 Fab. AHC: Anti-human IgG Fc Capture biosensors used to load and immobilize C68.61 IgG. SAX: Streptavidin biosensors used to load and immobilize biotinylated S-6P trimers for detecting soluble C68.61 IgG and Fab.
(TIF)

S7 Fig RBD structural orders of mosaic OX heterotrimer from competitive BLI. Omicron, OX and XBB S-6P trimers interact with C68.13 IgG to saturate RBDs on the Omicron protomers. Additional hACE2 competes binding to the available RBDs on the XBB protomers, indicating RBD structural orders are retained in the mosaic heterotrimers. Both association phases are 360 seconds.
(TIF)

S1 Table Experimental yields (in mg) of protein preparations from one liter of Expi293F cell culture. (PDF)

S2 Table DLS measurements of five spike trimers and polydispersity (PD). (PDF)

S3 Table Statistics reporting significant differences in HDX-MS uptake levels from the comparisons in Fig 2. (PDF)

S4 Table BLI binding kinetics data for Figs 6 and S6. (PDF)

## References

[1] Harvey WT, Carabelli AM, Jackson B, Gupta RK, Thomson EC, Harrison EM, et al. SARS-CoV-2 variants, spike mutations and immune escape. Nat Rev Microbiol. 2021;19(7):409–24. doi: 10.1038/s41579-021-00573-0 34075212 PMC8167834

[2] Markov PV, Ghafari M, Beer M, Lythgoe K, Simmonds P, Stilianakis NI, et al. The evolution of SARS-CoV-2. Nat Rev Microbiol. 2023;21(6):361–79. doi: 10.1038/s41579-023-00878-2 37020110

[3] Zhou P, Yang X-L, Wang X-G, Hu B, Zhang L, Zhang W, et al. A pneumonia outbreak associated with a new coronavirus of probable bat origin. Nature. 2020;579(7798):270–3. doi: 10.1038/s41586-020-2012-7 32015507 PMC7095418

[4] Walls AC, Park Y-J, Tortorici MA, Wall A, McGuire AT, Veesler D. Structure, Function, and Antigenicity of the SARS-CoV-2 Spike Glycoprotein. Cell. 2020;181(2):281-292.e6. doi: 10.1016/j.cell.2020.02.058 32155444 PMC7102599

[5] Shang J, Wan Y, Luo C, Ye G, Geng Q, Auerbach A, et al. Cell entry mechanisms of SARS-CoV-2. Proc Natl Acad Sci U S A. 2020;117(21):11727–34. 32376634 10.1073/pnas.2003138117 PMC7260975

[6] Korber B, Fischer WM, Gnanakaran S, Yoon H, Theiler J, Abfalterer W, et al. Tracking Changes in SARS-CoV-2 Spike: Evidence that D614G Increases Infectivity of the COVID-19 Virus. Cell. 2020;182(4):812-827.e19. doi: 10.1016/j.cell.2020.06.043 32697968 PMC7332439

[7] Wrapp D, Wang N, Corbett KS, Goldsmith JA, Hsieh CL, Abiona O, et al. Cryo-EM structure of the 2019-nCoV spike in the prefusion conformation. Science. 2020;367(6483):1260–3. 32075877 10.1126/science.abb2507 PMC7164637

[8] Yurkovetskiy L, Wang X, Pascal KE, Tomkins-Tinch C, Nyalile TP, Wang Y, et al. Structural and Functional Analysis of the D614G SARS-CoV-2 Spike Protein Variant. Cell. 2020;183(3):739-751.e8. doi: 10.1016/j.cell.2020.09.032 32991842 PMC7492024

[9] Gobeil SM-C, Janowska K, McDowell S, Mansouri K, Parks R, Manne K, et al. D614G Mutation Alters SARS-CoV-2 Spike Conformation and Enhances Protease Cleavage at the S1/S2 Junction. Cell Rep. 2021;34(2):108630. doi: 10.1016/j.celrep.2020.108630 33417835 PMC7762703

[10] Benton DJ, Wrobel AG, Roustan C, Borg A, Xu P, Martin SR, et al. The effect of the D614G substitution on the structure of the spike glycoprotein of SARS-CoV-2. Proc Natl Acad Sci U S A. 2021;118(9). 10.1073/pnas.2022586118 PMC7936381 33579792

[11] Zhang J, Cai Y, Xiao T, Lu J, Peng H, Sterling SM, et al. Structural impact on SARS-CoV-2 spike protein by D614G substitution. Science. 2021;372(6541):525–30. 33727252 10.1126/science.abf2303 PMC8139424

[12] Díaz-Salinas MA, Li Q, Ejemel M, Yurkovetskiy L, Luban J, Shen K, et al. Conformational dynamics and allosteric modulation of the SARS-CoV-2 spike. Elife. 2022;11. 10.7554/eLife.75433 PMC8963877 35323111

[13] Chen C, Zhu R, Hodge EA, Díaz-Salinas MA, Nguyen A, Munro JB, et al. hACE2-Induced Allosteric Activation in SARS-CoV versus SARS-CoV-2 Spike Assemblies Revealed by Structural Dynamics. ACS Infect Dis. 2023;9(6):1180–9. doi: 10.1021/acsinfecdis.3c00010 37166130 PMC10228703

[14] Barnes CO, Jette CA, Abernathy ME, Dam K-MA, Esswein SR, Gristick HB, et al. SARS-CoV-2 neutralizing antibody structures inform therapeutic strategies. Nature. 2020;588(7839):682–7. doi: 10.1038/s41586-020-2852-1 33045718 PMC8092461

[15] Robbiani DF, Gaebler C, Muecksch F, Lorenzi JCC, Wang Z, Cho A, et al. Convergent antibody responses to SARS-CoV-2 in convalescent individuals. Nature. 2020;584(7821):437–42. doi: 10.1038/s41586-020-2456-9 32555388 PMC7442695

[16] Barnes CO, West AP Jr, Huey-Tubman KE, Hoffmann MAG, Sharaf NG, Hoffman PR, et al. Structures of Human Antibodies Bound to SARS-CoV-2 Spike Reveal Common Epitopes and Recurrent Features of Antibodies. Cell. 2020;182(4):828-842.e16. doi: 10.1016/j.cell.2020.06.025 32645326 PMC7311918

[17] Khan A, Zia T, Suleman M, Khan T, Ali SS, Abbasi AA, et al. Higher infectivity of the SARS-CoV-2 new variants is associated with K417N/T, E484K, and N501Y mutants: An insight from structural data. J Cell Physiol. 2021;236(10):7045–57. doi: 10.1002/jcp.30367 33755190 PMC8251074

[18] Volz E, Mishra S, Chand M, Barrett JC, Johnson R, Geidelberg L, et al. Assessing transmissibility of SARS-CoV-2 lineage B.1.1.7 in England. Nature. 2021;593(7858):266–9. 33767447 10.1038/s41586-021-03470-x

[19] Chen J, Wang R, Gilby NB, Wei GW. Omicron Variant (B.1.1.529): Infectivity, Vaccine Breakthrough, and Antibody Resistance. J Chem Inf Model. 2022;62(2):412–22. 34989238 10.1021/acs.jcim.1c01451 PMC8751645

[20] Chen J, Wei G-W. Omicron BA.2 (B.1.1.529.2): High Potential for Becoming the Next Dominant Variant. J Phys Chem Lett. 2022;13(17):3840–9. doi: 10.1021/acs.jpclett.2c00469 35467344 PMC9063109

[21] Tamura T, Ito J, Uriu K, Zahradnik J, Kida I, Anraku Y, et al. Virological characteristics of the SARS-CoV-2 XBB variant derived from recombination of two Omicron subvariants. Nat Commun. 2023;14(1):2800. doi: 10.1038/s41467-023-38435-3 37193706 PMC10187524

[22] He Q, Wu L, Xu Z, Wang X, Xie Y, Chai Y, et al. An updated atlas of antibody evasion by SARS-CoV-2 Omicron sub-variants including BQ.1.1 and XBB. Cell Rep Med. 2023;4(4):100991. doi: 10.1016/j.xcrm.2023.100991 37019110 PMC10027947

[23] Wang Q, Iketani S, Li Z, Liu L, Guo Y, Huang Y, et al. Alarming antibody evasion properties of rising SARS-CoV-2 BQ and XBB subvariants. Cell. 2023;186(2):279–86.e8. 36580913 10.1016/j.cell.2022.12.018 PMC9747694

[24] Uraki R, Ito M, Furusawa Y, Yamayoshi S, Iwatsuki-Horimoto K, Adachi E, et al. Humoral immune evasion of the omicron subvariants BQ.1.1 and XBB. Lancet Infect Dis. 2023;23(1):30–2. doi: 10.1016/S1473-3099(22)00816-7 36495917 PMC9729000

[25] Yue C, Song W, Wang L, Jian F, Chen X, Gao F, et al. ACE2 binding and antibody evasion in enhanced transmissibility of XBB.1.5. Lancet Infect Dis. 2023;23(3):278–80. doi: 10.1016/S1473-3099(23)00010-5 36746173 PMC9897732

[26] Polack FP, Thomas SJ, Kitchin N, Absalon J, Gurtman A, Lockhart S, et al. Safety and Efficacy of the BNT162b2 mRNA Covid-19 Vaccine. New Engl J Med. 2020;383(27):2603–15. 33301246 10.1056/NEJMoa2034577 PMC7745181

[27] Corbett KS, Edwards DK, Leist SR, Abiona OM, Boyoglu-Barnum S, Gillespie RA, et al. SARS-CoV-2 mRNA vaccine design enabled by prototype pathogen preparedness. Nature. 2020;586(7830):567–71. doi: 10.1038/s41586-020-2622-0 32756549 PMC7581537

[28] Martínez-Flores D, Zepeda-Cervantes J, Cruz-Reséndiz A, Aguirre-Sampieri S, Sampieri A, Vaca L. SARS-CoV-2 Vaccines Based on the Spike Glycoprotein and Implications of New Viral Variants. Front Immunol. 2021;12:701501. 34322129 10.3389/fimmu.2021.701501 PMC8311925

[29] Baden LR, El Sahly HM, Essink B, Kotloff K, Frey S, Novak R, et al. Efficacy and Safety of the mRNA-1273 SARS-CoV-2 Vaccine. N Engl J Med. 2021;384(5):403–16. doi: 10.1056/NEJMoa2035389 33378609 PMC7787219

[30] Yang J, Wang W, Chen Z, Lu S, Yang F, Bi Z, et al. A vaccine targeting the RBD of the S protein of SARS-CoV-2 induces protective immunity. Nature. 2020;586(7830):572–7. 32726802 10.1038/s41586-020-2599-8

[31] Walls AC, Fiala B, Schäfer A, Wrenn S, Pham MN, Murphy M, et al. Elicitation of Potent Neutralizing Antibody Responses by Designed Protein Nanoparticle Vaccines for SARS-CoV-2. Cell. 2020;183(5):1367-1382.e17. doi: 10.1016/j.cell.2020.10.043 33160446 PMC7604136

[32] Pardi N, Hogan MJ, Porter FW, Weissman D. mRNA vaccines - a new era in vaccinology. Nat Rev Drug Discov. 2018;17(4):261–79. doi: 10.1038/nrd.2017.243 29326426 PMC5906799

[33] Nance KD, Meier JL. Modifications in an Emergency: The Role of N1-Methylpseudouridine in COVID-19 Vaccines. ACS Cent Sci. 2021;7(5):748–56. doi: 10.1021/acscentsci.1c00197 34075344 PMC8043204

[34] Anderson EJ, Rouphael NG, Widge AT, Jackson LA, Roberts PC, Makhene M, et al. Safety and Immunogenicity of SARS-CoV-2 mRNA-1273 Vaccine in Older Adults. N Engl J Med. 2020;383(25):2427–38. doi: 10.1056/NEJMoa2028436 32991794 PMC7556339

[35] Thomas SJ, Moreira ED Jr, Kitchin N, Absalon J, Gurtman A, Lockhart S, et al. Safety and Efficacy of the BNT162b2 mRNA Covid-19 Vaccine through 6 Months. N Engl J Med. 2021;385(19):1761–73. doi: 10.1056/NEJMoa2110345 34525277 PMC8461570

[36] Sahin U, Muik A, Vogler I, Derhovanessian E, Kranz LM, Vormehr M, et al. BNT162b2 vaccine induces neutralizing antibodies and poly-specific T cells in humans. Nature. 2021;595(7868):572–7. doi: 10.1038/s41586-021-03653-6 34044428

[37] Chalkias S, Harper C, Vrbicky K, Walsh SR, Essink B, Brosz A, et al. A bivalent omicron-containing booster vaccine against Covid-19. New Engl J Med. 2022;387(14):1279–91. 36112399 10.1056/NEJMoa2208343 PMC9511634

[38] Tseng HF, Ackerson BK, Sy LS, Tubert JE, Luo Y, Qiu S, et al. mRNA-1273 bivalent (original and Omicron) COVID-19 vaccine effectiveness against COVID-19 outcomes in the United States. Nat Commun. 2023;14(1):5851. doi: 10.1038/s41467-023-41537-7 37730701 PMC10511551

[39] Cohen AA, Gnanapragasam PNP, Lee YE, Hoffman PR, Ou S, Kakutani LM, et al. Mosaic nanoparticles elicit cross-reactive immune responses to zoonotic coronaviruses in mice. Science. 2021;371(6530):735–41. doi: 10.1126/science.abf6840 33436524 PMC7928838

[40] Arevalo CP, Bolton MJ, Le Sage V, Ye N, Furey C, Muramatsu H, et al. A multivalent nucleoside-modified mRNA vaccine against all known influenza virus subtypes. Science. 2022;378(6622):899–904. doi: 10.1126/science.abm0271 36423275 PMC10790309

[41] Cankat S, Demael MU, Swadling L. In search of a pan-coronavirus vaccine: next-generation vaccine design and immune mechanisms. Cell Mol Immunol. 2024;21(2):103–18. 38148330 10.1038/s41423-023-01116-8 PMC10805787

[42] Hsieh C-L, Goldsmith JA, Schaub JM, DiVenere AM, Kuo H-C, Javanmardi K, et al. Structure-based design of prefusion-stabilized SARS-CoV-2 spikes. Science. 2020;369(6510):1501–5. doi: 10.1126/science.abd0826 32703906 PMC7402631

[43] Ashkenazy H, Abadi S, Martz E, Chay O, Mayrose I, Pupko T, et al. ConSurf 2016: an improved methodology to estimate and visualize evolutionary conservation in macromolecules. Nucleic Acid Res. 2016;44(W1):W344–50. 10.1093/nar/gkw408 PMC4987940 27166375

[44] Kwong PD, Mascola JR. HIV-1 vaccines based on antibody identification, B cell ontogeny, and epitope structure. Immunity. 2018;48(5):855–71. 29768174 10.1016/j.immuni.2018.04.029

[45] Reily C, Stewart TJ, Renfrow MB, Novak J. Glycosylation in health and disease. Nat Rev Nephrol. 2019;15(6):346–66. doi: 10.1038/s41581-019-0129-4 30858582 PMC6590709

[46] Sztain T, Ahn S-H, Bogetti AT, Casalino L, Goldsmith JA, Seitz E, et al. A glycan gate controls opening of the SARS-CoV-2 spike protein. Nat Chem. 2021;13(10):963–8. doi: 10.1038/s41557-021-00758-3 34413500 PMC8488004

[47] Watanabe Y, Allen JD, Wrapp D, McLellan JS, Crispin M. Site-specific glycan analysis of the SARS-CoV-2 spike. Science. 2020;369(6501):330–3. doi: 10.1126/science.abb9983 32366695 PMC7199903

[48] Brouwer PJM, Brinkkemper M, Maisonnasse P, Dereuddre-Bosquet N, Grobben M, Claireaux M, et al. Two-component spike nanoparticle vaccine protects macaques from SARS-CoV-2 infection. Cell. 2021;184(5):1188-1200.e19. doi: 10.1016/j.cell.2021.01.035 33577765 PMC7834972

[49] Calvaresi V, Wrobel AG, Toporowska J, Hammerschmid D, Doores KJ, Bradshaw RT, et al. Structural dynamics in the evolution of SARS-CoV-2 spike glycoprotein. Nat Commun. 2023;14(1):1421. doi: 10.1038/s41467-023-36745-0 36918534 PMC10013288

[50] Starr TN, Czudnochowski N, Liu Z, Zatta F, Park Y-J, Addetia A, et al. SARS-CoV-2 RBD antibodies that maximize breadth and resistance to escape. Nature. 2021;597(7874):97–102. doi: 10.1038/s41586-021-03807-6 34261126 PMC9282883

[51] Wang Y, Yan A, Song D, Duan M, Dong C, Chen J, et al. Identification of a highly conserved neutralizing epitope within the RBD region of diverse SARS-CoV-2 variants. Nat Commun. 2024;15(1):842. doi: 10.1038/s41467-024-45050-3 38287016 PMC10825162

[52] Li L, Liao H, Meng Y, Li W, Han P, Liu K, et al. Structural basis of human ACE2 higher binding affinity to currently circulating Omicron SARS-CoV-2 sub-variants BA.2 and BA.1.1. Cell. 2022;185(16):2952-2960.e10. doi: 10.1016/j.cell.2022.06.023 35809570 PMC9212699

[53] Guenthoer J, Lilly M, Starr TN, Dadonaite B, Lovendahl KN, Croft JT, et al. Identification of broad, potent antibodies to functionally constrained regions of SARS-CoV-2 spike following a breakthrough infection. Proc Natl Acad Sci U S A. 2023;120(23):e2220948120. doi: 10.1073/pnas.2220948120 37253011 PMC10265947

[54] Wells HL, Bonavita CM, Navarrete-Macias I, Vilchez B, Rasmussen AL, Anthony SJ. The coronavirus recombination pathway. Cell Host Microbe. 2023;31(6):874–89. doi: 10.1016/j.chom.2023.05.003 37321171 PMC10265781

[55] Sabir JSM, Lam TT-Y, Ahmed MMM, Li L, Shen Y, Abo-Aba SEM, et al. Co-circulation of three camel coronavirus species and recombination of MERS-CoVs in Saudi Arabia. Science. 2016;351(6268):81–4. doi: 10.1126/science.aac8608 26678874

[56] Gussow AB, Auslander N, Faure G, Wolf YI, Zhang F, Koonin EV. Genomic determinants of pathogenicity in SARS-CoV-2 and other human coronaviruses. Proc Natl Acad Sci. 2020;117(26):15193–9. 32522874 10.1073/pnas.2008176117 PMC7334499

[57] Redd AD, Quinn TC, Tobian AA. Frequency and implications of HIV superinfection. Lancet Infect Dis. 2013;13(7):622–8. doi: 10.1016/S1473-3099(13)70066-5 23726798 PMC3752600

[58] Dadonaite B, Brown J, McMahon TE, Farrell AG, Figgins MD, Asarnow D, et al. Spike deep mutational scanning helps predict success of SARS-CoV-2 clades. Nature. 2024;631(8021):617–26. 38961298 10.1038/s41586-024-07636-1 PMC11254757

[59] Cui Z, Liu P, Wang N, Wang L, Fan K, Zhu Q, et al. Structural and functional characterizations of infectivity and immune evasion of SARS-CoV-2 Omicron. Cell. 2022;185(5):860-871.e13. doi: 10.1016/j.cell.2022.01.019 35120603 PMC8786603

[60] Gobeil SM-C, Henderson R, Stalls V, Janowska K, Huang X, May A, et al. Structural diversity of the SARS-CoV-2 Omicron spike. Mol Cell. 2022;82(11):2050-2068.e6. doi: 10.1016/j.molcel.2022.03.028 35447081 PMC8947964

[61] Cohen AA, van Doremalen N, Greaney AJ, Andersen H, Sharma A, Starr TN, et al. Mosaic RBD nanoparticles protect against challenge by diverse sarbecoviruses in animal models. Science. 2022;377(6606):eabq0839. doi: 10.1126/science.abq0839 35857620 PMC9273039

[62] Hills RA, Tan TK, Cohen AA, Keeffe JR, Keeble AH, Gnanapragasam PNP, et al. Proactive vaccination using multiviral Quartet Nanocages to elicit broad anti-coronavirus responses. Nat Nanotechnol. 2024;19(8):1216–23. doi: 10.1038/s41565-024-01655-9 38710880 PMC11329374

[63] Wang E, Cohen AA, Caldera LF, Keeffe JR, Rorick AV, Adia YM, et al. Designed mosaic nanoparticles enhance cross-reactive immune responses in mice. Cell. 2025;188(4):1036–50.e11. doi: 10.1016/j.cell.2024.12.015 39855201 PMC11845252

[64] Dosey A, Ellis D, Boyoglu-Barnum S, Syeda H, Saunders M, Watson MJ, et al. Combinatorial immune refocusing within the influenza hemagglutinin RBD improves cross-neutralizing antibody responses. Cell Rep. 2023;42(12):113553. doi: 10.1016/j.celrep.2023.113553 38096052 PMC10801708

[65] Pallesen J, Wang N, Corbett KS, Wrapp D, Kirchdoerfer RN, Turner HL, et al. Immunogenicity and structures of a rationally designed prefusion MERS-CoV spike antigen. Proc Natl Acad Sci U S A. 2017;114(35):E7348–57. doi: 10.1073/pnas.1707304114 28807998 PMC5584442

[66] Watson MJ, Harkewicz R, Hodge EA, Vorauer C, Palmer J, Lee KK, et al. Simple Platform for Automating Decoupled LC-MS Analysis of Hydrogen/Deuterium Exchange Samples. J Am Soc Mass Spectrom. 2021;32(2):597–600. doi: 10.1021/jasms.0c00341 33284630 PMC7863070

