# Supplementary figures and images for: Interprotomer crosstalk in mosaic viral glycoprotein trimers provides insight into polyvalent immunogen co-assembly
Source: PLoS Pathog. 2025 Sep 22;21(9):e1013143. doi: 10.1371/journal.ppat.1013143 (PMC12483203; doi:10.1371/journal.ppat.1013143)

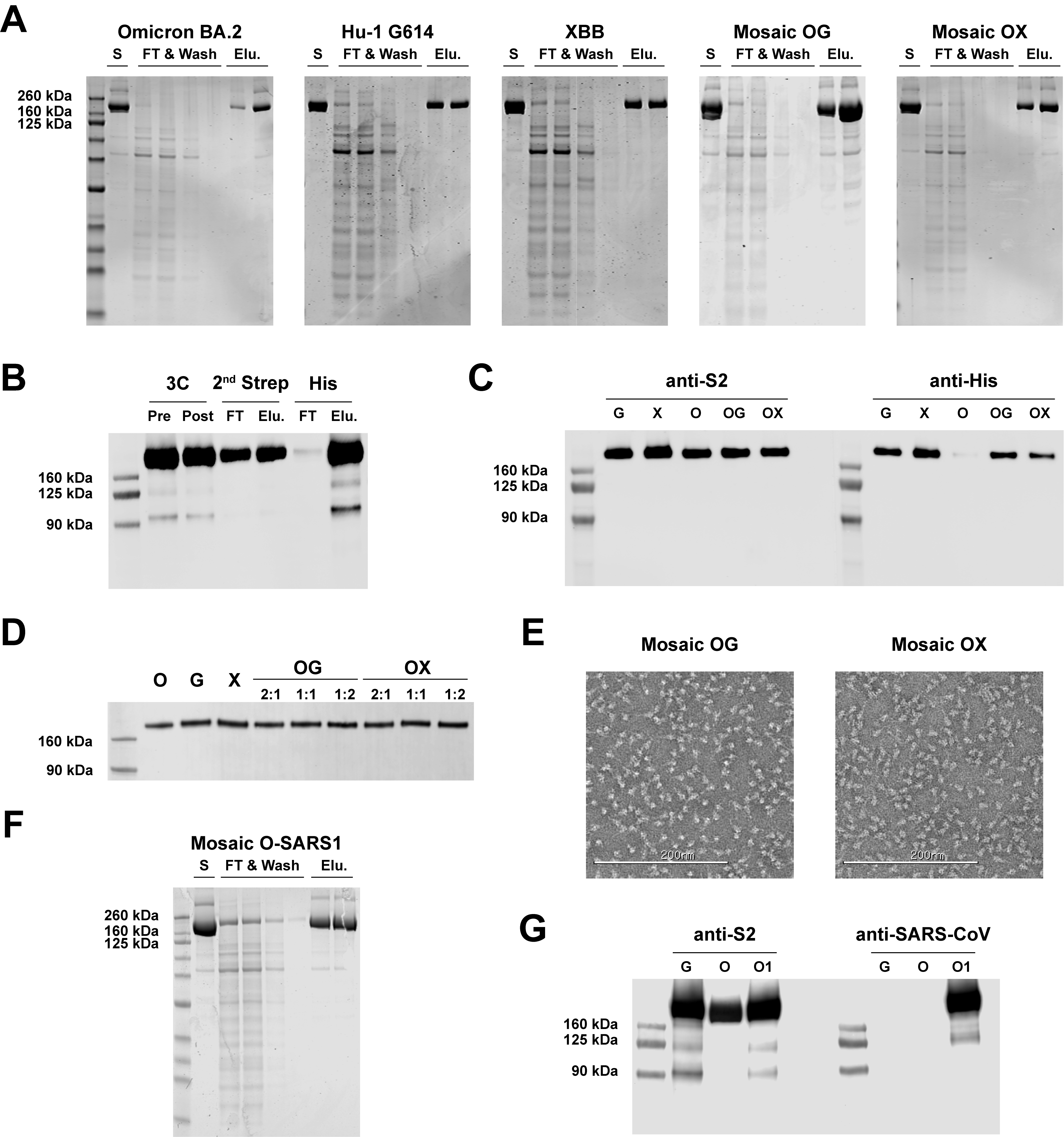

Supplement: S1 Fig — (A) SDS-PAGE gels with Coomassie blue staining from affinity purification of each S trimer. FT: Flow-through; Elu.: Elution (B) Western blot with anti-His primary antibody indicates the efficiency of HRV 3C protease digestion and tandem-affinity purifications in separating mosaic heterotrimers from homotrimers. (C) Western blot characterization and quantification of homotrimers and heterotrimers using anti-S2-subunit and anti-His primary antibodies. (D) Western blot quantification of the total S expression from different co-transfection ratios. (E) Negative-stain electron microscopy images illustrate the morphology of Omicron-G614 heterotrimer and Omicron-XBB heterotrimer. (F) SDS-PAGE gel with Coomassie blue staining from affinity purification of Omicron-SARS-CoV (O-SARS1) S trimer. (G) Western blot characterization of homotrimers and heterotrimers using anti-S2-subunit and anti-SARS-CoV primary antibodies. O1: O-SARS1 mosaic heterotrimer. This figure is related to Fig 1. (TIF) [file ppat.1013143.s001.tif]

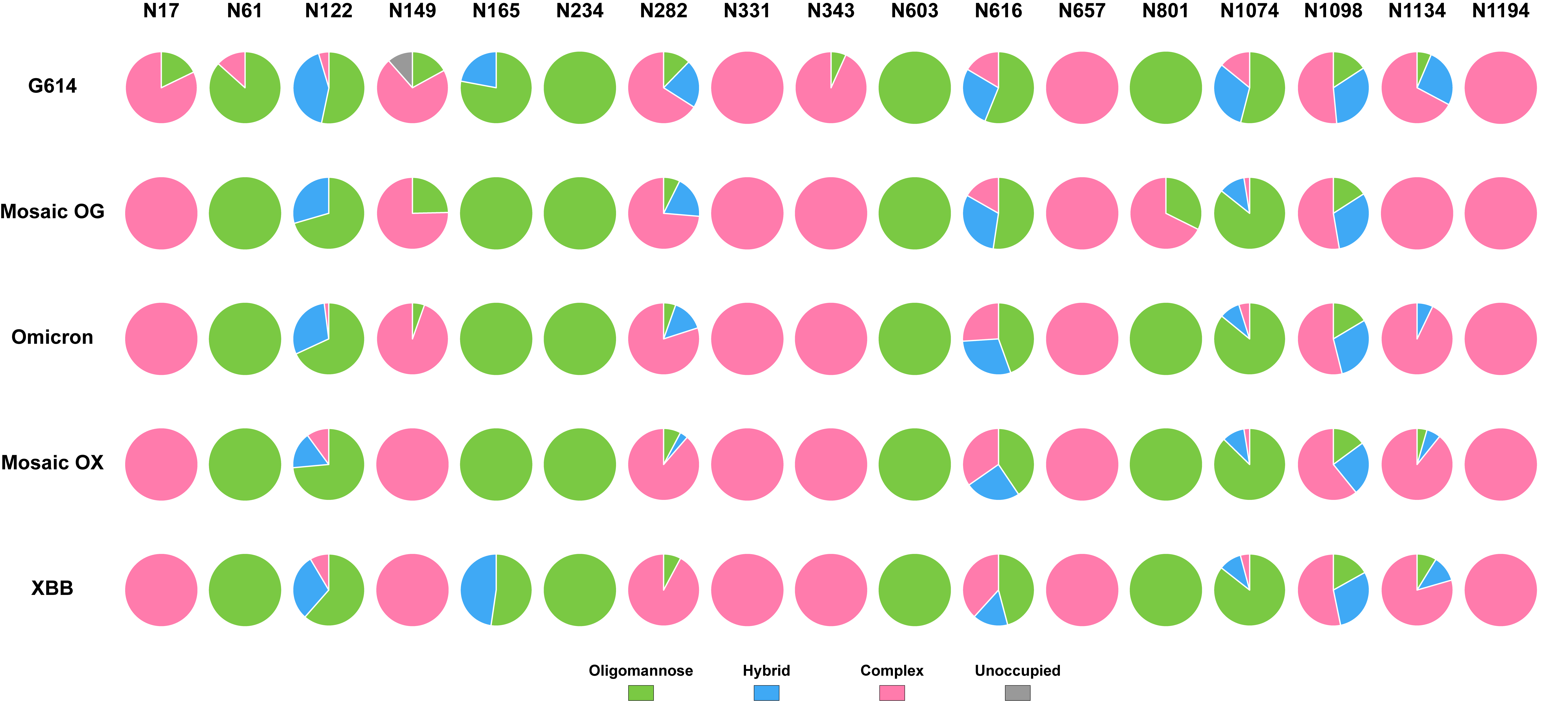

Supplement: S2 Fig — 17 out of 22 N-glycosylation sites are characterized and compared. This figure is related to Fig 1F. (TIF) [file ppat.1013143.s002.tif]

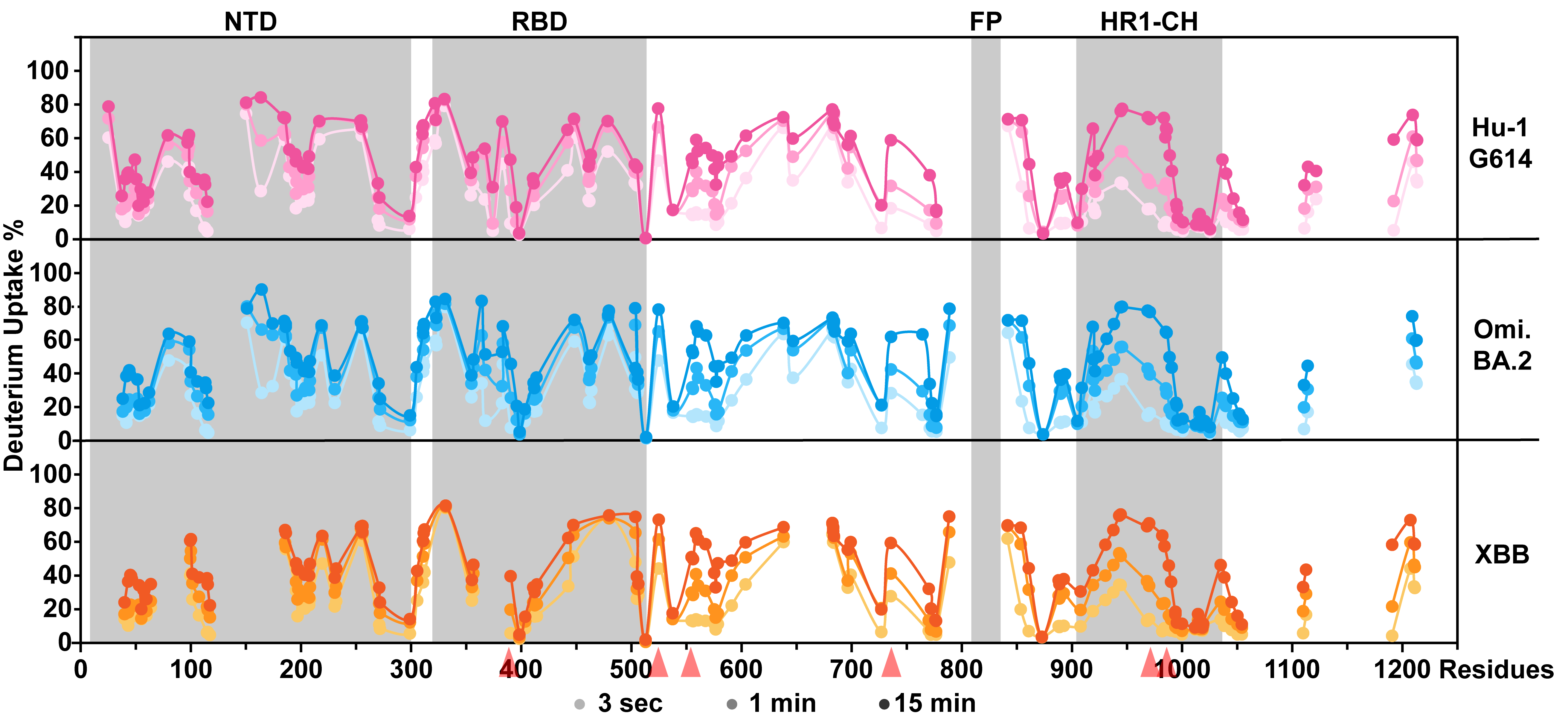

Supplement: S3 Fig — Butterfly deuterium uptake plots depict strain-specific S dynamic profiles with highlights in the functionally important domains. This figure is related to Fig 2. (TIF) [file ppat.1013143.s003.tif]

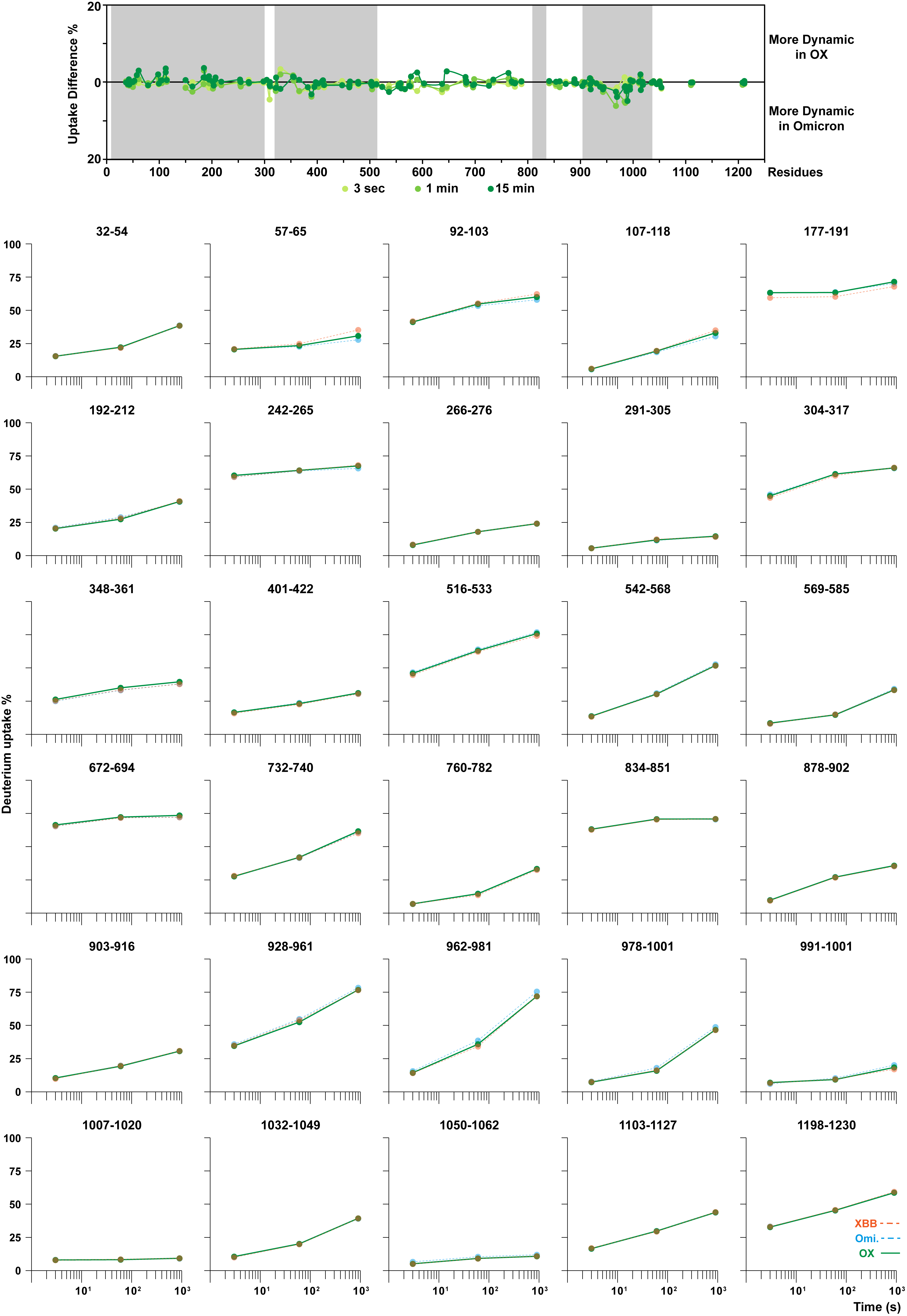

Supplement: S4 Fig — This figure is related to Fig 3. (TIF) [file ppat.1013143.s004.tif]

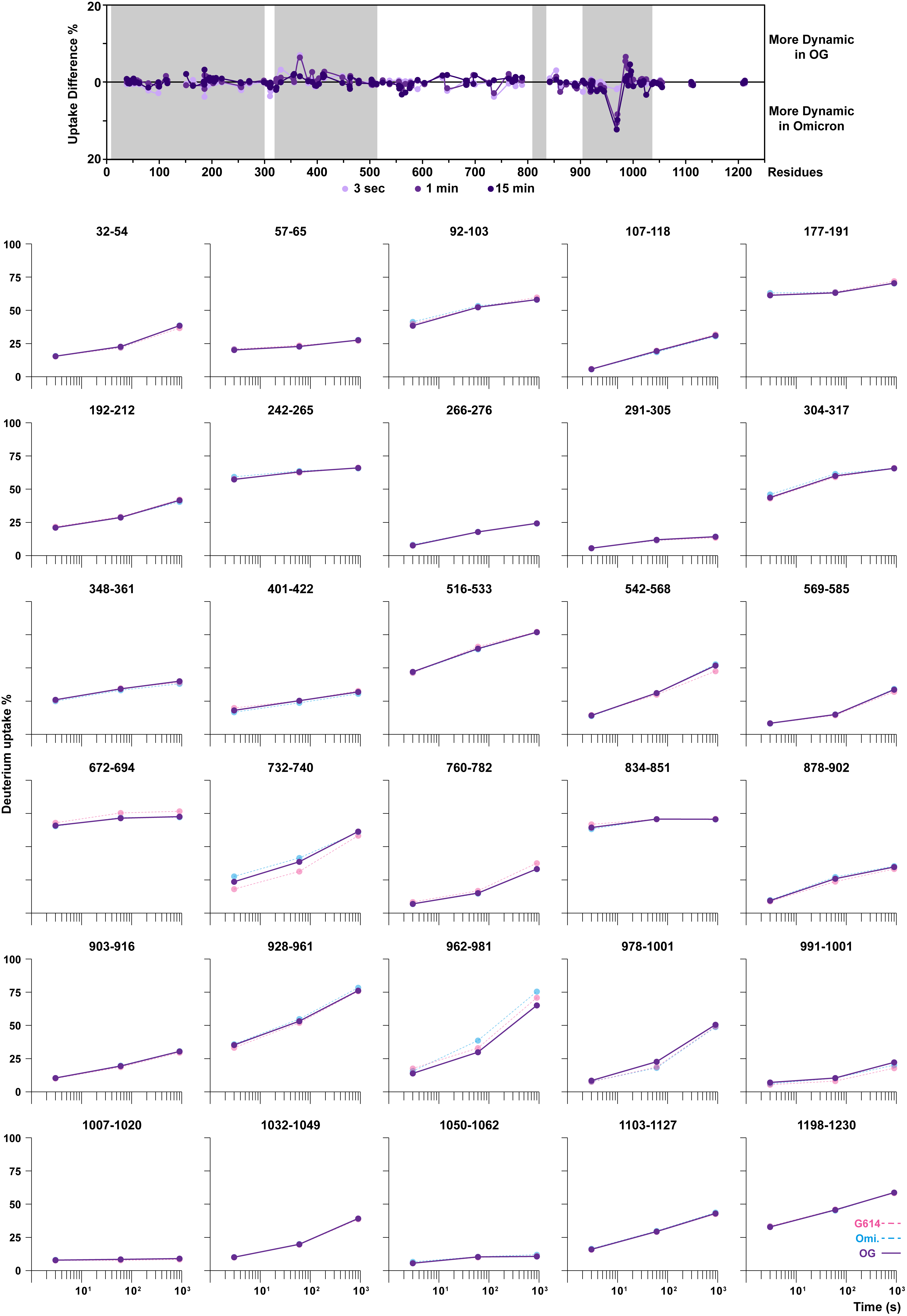

Supplement: S5 Fig — This figure is related to Fig 4. (TIF) [file ppat.1013143.s005.tif]

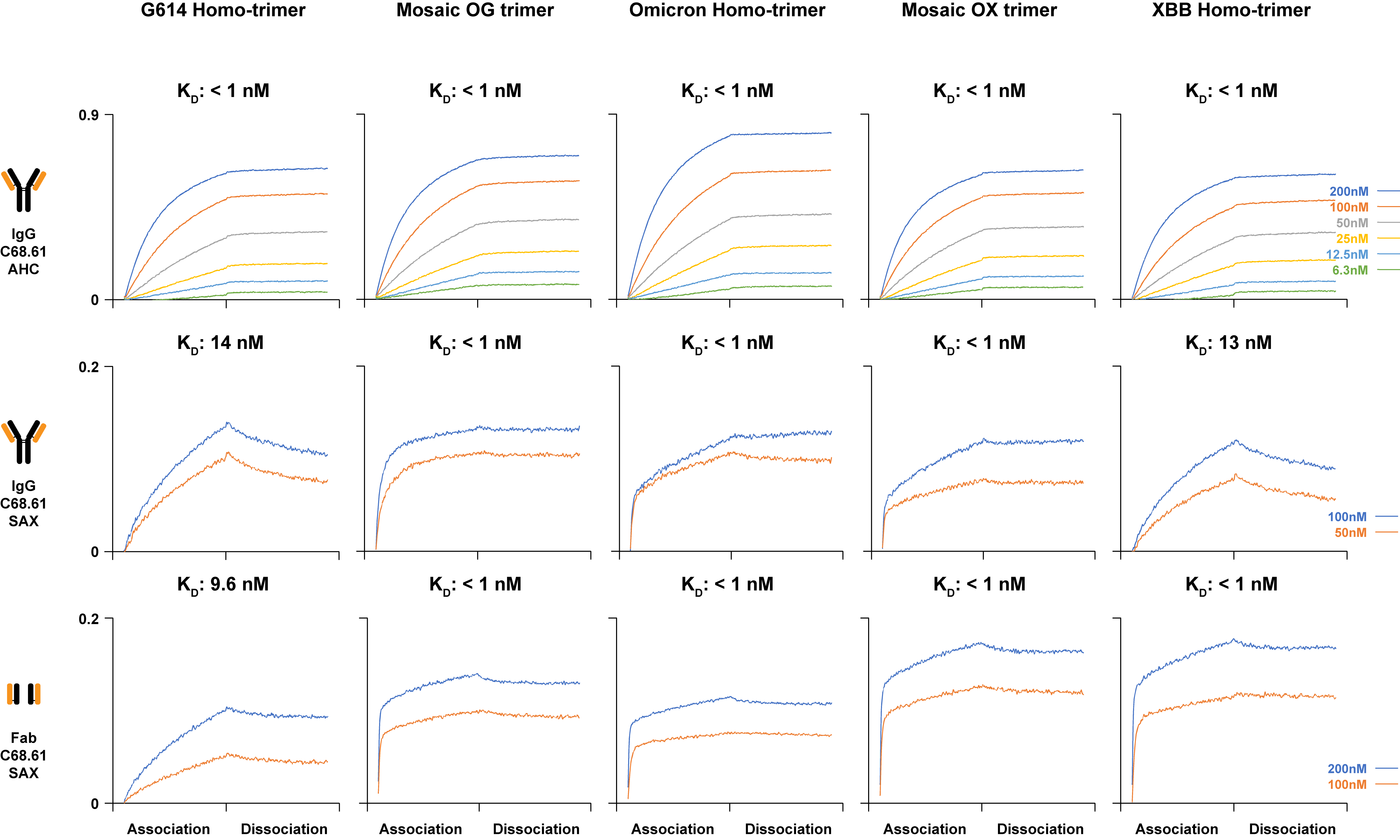

Supplement: S6 Fig — BLI measurements on five biotinylated S-6P trimers interacting with C68.61 IgG and C68.61 Fab. AHC: Anti-human IgG Fc Capture biosensors used to load and immobilize C68.61 IgG. SAX: Streptavidin biosensors used to load and immobilize biotinylated S-6P trimers for detecting soluble C68.61 IgG and Fab. (TIF) [file ppat.1013143.s006.tif]

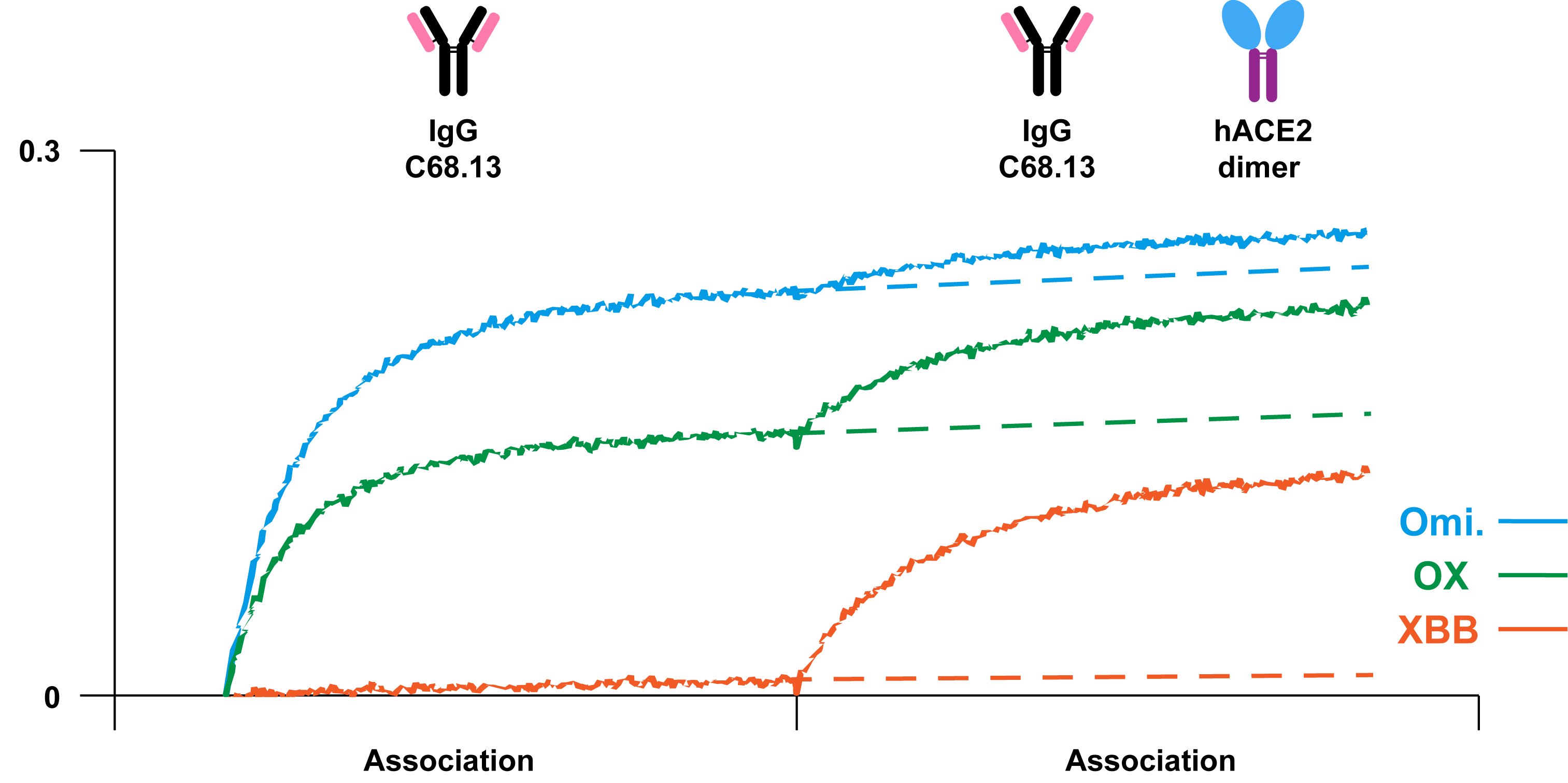

Supplement: S7 Fig — Omicron, OX and XBB S-6P trimers interact with C68.13 IgG to saturate RBDs on the Omicron protomers. Additional hACE2 competes binding to the available RBDs on the XBB protomers, indicating RBD structural orders are retained in the mosaic heterotrimers. Both association phases are 360 seconds. (TIF) [file ppat.1013143.s007.tif]
