# Supplementary material for: Interprotomer crosstalk in mosaic viral glycoprotein trimers provides insight into polyvalent immunogen co-assembly
Source: PLoS Pathog. 2025 Sep 22;21(9):e1013143. doi: 10.1371/journal.ppat.1013143 (PMC12483203; doi:10.1371/journal.ppat.1013143)
Supplement: S1 Table — (PDF) [file ppat.1013143.s008.pdf]

S1 Table. Experimental yields (in mg) of protein preparations from one liter of Expi293F cell culture.

Spike Constructs	1st anti-Strep Column	3C Digestion & 2nd anti-Strep Column	anti-His Column[*]
G614	-	-	6.8[II]
Omicron	4.4[I], 10.0[II]	2.3[I], 7.7[II]	-
XBB	-	-	5.8[II]
OG	4.4[I], 10.8[II]	2.5[I], 8.4[II]	(1.5 + 0.5)[I] (4.7 + 1.9)[II]
OX	3.4[I], 9.6[II]	1.7[I], 6.0[II]	(1.2 + 0.4)[I] (3.6 + 1.2)[II]
O-SARS1[III]	9.8	6.4	(3.6 + 1.0)

Strep: affinity chromatography against Twin-Strep tag; 3C: HRV 3C protease digestion; His: affinity chromatography against Poly-His tag.

[*] Yields of OG, OX and O-SARS1 are shown in the form of mosaic heterotrimer mass from His-tag affinity purification elution + Omicron homotrimer mass from flow-through.

[I] Parallel preparations of Omicron, OG and OX from high-passage Expi293F cell (Sep-Nov 2023).

[II] Parallel preparations of G614, Omicron, XBB, OG and OX from low-passage Expi293F cell (Jan-Feb 2024).

[III] Preparation of O-SARS1 from low-passage Expi293F cell (Jun-Jul 2025).
