# Supplementary material for: Interprotomer crosstalk in mosaic viral glycoprotein trimers provides insight into polyvalent immunogen co-assembly
Source: PLoS Pathog. 2025 Sep 22;21(9):e1013143. doi: 10.1371/journal.ppat.1013143 (PMC12483203; doi:10.1371/journal.ppat.1013143)
Supplement: S2 Table — (PDF) [file ppat.1013143.s009.pdf]

S2 Table. DLS measurements of five spike trimers and polydispersity (PD).

Spike	Radius (nm)	PD (%)
G614	10.72±0.13	14.1±2.1
Omicron	11.70±0.12	17.1±2.4
XBB	11.60±0.08	15.1±1.8
OG	10.74±0.21	16.1±4.8
OX	10.71±0.14	12.8±1.8
