# Supplementary material for: Interprotomer crosstalk in mosaic viral glycoprotein trimers provides insight into polyvalent immunogen co-assembly
Source: PLoS Pathog. 2025 Sep 22;21(9):e1013143. doi: 10.1371/journal.ppat.1013143 (PMC12483203; doi:10.1371/journal.ppat.1013143)
Supplement: S3 Table — (PDF) [file ppat.1013143.s010.pdf]

S3 Table. Statistics reporting significant differences in HDX-MS uptake levels from the comparisons in Fig 2.

Peptide	Comparison	Time Point	Effect Size	p Value
388-392	G614 vs. Omi.	1 min	4.07	0.002
	XBB vs. Omi.	1 min	4.93	< 0.001
	XBB vs. Omi.	15 min	4.74	0.004
982-990	XBB vs. Omi.	1 min	6.48	0.002
516-533	G614 vs. Omi.	1 min	2.00	0.005
542-568	G614 vs. Omi.	15 min	5.31	< 0.001
962-981	G614 vs. Omi.	1 min	5.69	0.007
	G614 vs. Omi.	15 min	4.61	0.003
	XBB vs. Omi.	1 min	4.48	0.018
	XBB vs. Omi.	15 min	3.52	0.035
732-740	G614 vs. Omi.	3 sec	9.54	< 0.001
	G614 vs. Omi.	1 min	10.28	< 0.001
