# Supplementary material for: Interprotomer crosstalk in mosaic viral glycoprotein trimers provides insight into polyvalent immunogen co-assembly
Source: PLoS Pathog. 2025 Sep 22;21(9):e1013143. doi: 10.1371/journal.ppat.1013143 (PMC12483203; doi:10.1371/journal.ppat.1013143)
Supplement: S4 Table — (PDF) [file ppat.1013143.s011.pdf]

S4 Table. BLI binding kinetics data for Figs 6 and S6.

Binder		G614	OG	Omicron	OX	XBB
hACE2 dimer	K_D (nM)	9.5	6.4	7.4	9.5	10
(AHC)	k_{on} ($M^{-1}s^{-1}$)	2.3×10^5	2.2×10^5	1.5×10^5	1.3×10^5	1.0×10^5
	k_{off} (s^{-1})	2.2×10^{-3}	1.4×10^{-3}	1.1×10^{-3}	1.2×10^{-3}	1.0×10^{-3}
c68.13 IgG		< 1	1.1	2.1	6.5	95
(AHC)		2.0×10^5	1.9×10^5	1.0×10^5	5.7×10^4	1.6×10^4
		2.7×10^{-5}	2.1×10^{-4}	2.2×10^{-4}	3.7×10^{-4}	1.5×10^{-3}
c68.13 IgG		1.1	1.1	1.4	6.5	52
(SAX)		4.3×10^5	3.6×10^5	3.3×10^5	3.3×10^5	1.2×10^5
		4.7×10^{-4}	3.9×10^{-4}	4.4×10^{-4}	2.1×10^{-3}	6.0×10^{-3}
c68.61 IgG		< 1	< 1	< 1	< 1	< 1
(AHC)		8.3×10^4	9.3×10^4	8.4×10^4	9.3×10^4	8.4×10^4
		ND	ND	ND	ND	ND
c68.61 IgG		14	< 1	< 1	< 1	13
(SAX)		1.0×10^5	4.8×10^5	4.4×10^5	4.4×10^5	1.3×10^5
		1.5×10^{-3}	ND	ND	ND	1.7×10^{-3}
c68.61 Fab		9.6	< 1	< 1	< 1	< 1
(SAX)		4.4×10^4	ND	ND	ND	ND
		4.3×10^{-4}	2.5×10^{-4}	ND	5.1×10^{-4}	ND

AHC: Anti-human IgG Fc Capture Biosensors used to load and immobilize hACE2-Fc dimer and IgGs for detecting soluble S-6P trimers.

SAX: Streptavidin Biosensors used to load and immobilize biotinylated S-6P trimers for detecting soluble IgGs and Fab.

ND: Not determined. The fitting curves could not reflect accurate k_{on} and k_{off} when fast-saturation ($k_{on} > 10^6$) or slow dissociation ($k_{off} < 10^{-5}$) occurred.
